# Nabiximols in Multiple Sclerosis: Beyond Spasticity—An Exploratory Systematic Review and Meta-Analysis of Symptomatic Outcomes

**DOI:** 10.3390/medsci14030346

**Published:** 2026-06-25

**Authors:** Dénes Kleiner, István László Horváth, Dóra Mátis, Rita Nagy, Dorottya Gergő, Katalin Lugosi, Gábor Fazekas, Péter Fehérvári, Péter Hegyi, Dezső Csupor

**Affiliations:** 1 University Pharmacy, Department of Pharmacy Administration, Semmelweis University, Hőgyes Endre utca 7-9., 1092 Budapest, Hungary; 2 Centre for Translational Medicine, Semmelweis University, Baross utca 22, 1085 Budapest, Hungary; 3 Heim Pál National Pediatric Institute, 1089 Budapest, Hungary; 4 Department of Pharmacognosy, Semmelweis University, Üllői út 26., 1085 Budapest, Hungary; 5 Multiple Sclerosis Centre, Bajcsy-Zsilinszky Hospital, Maglódi út 89-91, 1106 Budapest, Hungary; 6 Rehabilitation Clinic, Semmelweis University, Szanatórium utca 19, 1121 Budapest, Hungary; 7 Department of Rehabilitation, University of Szeged, Tisza Lajos körút 97, 6722 Szeged, Hungary; 8 Budapest Department of Biostatistics, University of Veterinary Medicine, István utca 2., 1078 Budapest, Hungary; 9 Institute for Translational Medicine, Medical School, University of Pécs, Szigeti út 12, 7624 Pécs, Hungary; 10 Institute of Pancreatic Diseases, Semmelweis University, Tömő utca 25-29, 1083 Budapest, Hungary; 11 Institute of Clinical Pharmacy, University of Szeged, Szikra utca 8, 6725 Szeged, Hungary

**Keywords:** Sativex, THC/CBD, spasms, bladder function, sleep disruption, multiple sclerosis

## Abstract

**Background/Objectives:** Nabiximols, a standardized extract of *Cannabis sativa*, has been approved as an add-on therapy for patients with moderate to severe spasticity associated with multiple sclerosis (MS). Moreover, current Italian treatment algorithms suggest that cannabis-based therapies may have further relevance in the management of MS. The aim of our systematic review and meta-analysis was to assess the effectiveness of nabiximols in relieving symptoms other than spasticity in adult MS patients. **Methods:** A systematic search was conducted in Web of Science, MEDLINE (via PubMed), Cochrane CENTRAL and Embase on September 10, 2025. Study selection was performed according to the predefined PROSPERO protocol (CRD42022329952). Data were combined into a common denominator and examined using a random-effects model with meta-regression expressed as mean difference (MD) and a 95% confidence interval (CI). Risk of bias was assessed using the Cochrane risk of bias instrument (RoB2) and the Risk Of Bias In Non-randomized Studies of Interventions (ROBINS-I) tool. **Results:** Of the 49 eligible articles, 25 were included in the statistical analysis (2949 patients). Significant improvements in spasm quality (MD = −16.87; 95% CI = (−29.75)–(−3.99)), bladder function (MD = −16.38; 95% CI = (−22.18)–(−10.58)), sleep disruption (MD = −15.75; 95% CI = (−22.02)–(−9.49)) and gait function (timed walk MD(s) = −5.31; 95% CI(s) = (−9.88)–(−0.74)) were observed. Time-dependency was not significant. The subject global impression of change (SGIC) improved significantly after the first month (odds ratio (OR) = 1.69; 95% CI = (1.30)–(2.18)). **Conclusion:** Beyond spasticity, nabiximols may represent a signal of benefit for bladder function, sleep disruption, spasm quality, and gait function in MS, in line with the “spasticity-plus” concept. However, the evidence certainty was low to very low, and these findings should be considered exploratory.

## 1. Introduction

Currently, an estimated 2.8 million people worldwide are living with multiple sclerosis (MS) [1]. MS remains incurable, with disease-modifying therapies capable only of slowing disease progression. Additional symptomatic treatments are often needed over time, but the symptoms do not respond well to existing therapies, as evidenced by the numerous off-label and unapproved therapeutic approaches [2,3]. Furthermore, the concurrent use of several medications increases the risk of adverse effects and drug interactions [4,5]. Therefore, clinicians need novel therapeutic strategies to address MS-associated symptoms [6].

In recent years, the concept of “spasticity-plus syndrome” has emerged in the symptomatic management of MS [4]. According to this concept, spasticity is not an isolated symptom, but is associated with several interrelated (or partly interrelated) problems, including sleep disruption, pain, fatigue, impaired bladder function and impaired gait function. This raises the possibility that targeting a common pathway, namely the cannabinoid system, may alleviate multiple symptoms simultaneously. The concept is also reflected in a recently proposed Italian treatment algorithm for MS management [7].

Nabiximols is a well-characterized extract of *Cannabis sativa* (27 mg/mL tetrahydrocannabinol (THC) and 25 mg/mL cannabidiol (CBD)) that acts on the cannabinoid system. It is effective in treatment-resistant MS-related spasticity and may also improve several associated symptoms. Moreover, nabiximols has shown encouraging results in the treatment of various symptoms such as pain, gait function, sleep disruption, bladder function, fatigue, and tremors [4,8].

However, comprehensive literature reviews have focused mainly on spasticity and pain, while the impact on other symptoms has been mostly overlooked [9,10,11]. Therefore, the primary question of our exploratory study was whether nabiximols improved MS-related symptoms other than spasticity and pain. To our knowledge, this is the first meta-analysis that quantitatively evaluates the effects of nabiximols across multiple MS-related symptom domains, including spasm quality, urinary dysfunction, sleep disruption and gait function; and incorporates temporal meta-regression to assess both the improvement and persistence of treatment effects. Compared with our previous work, which primarily focused on spasticity in comparative analyses, the present study evaluates proportional change across multiple symptom domains [8].

## 2. Materials and Methods

### 2.1. Search Strategy

This review was performed in accordance with the PRISMA (Preferred Reporting Items for Systematic Reviews and Meta-Analyses) guidelines and the Cochrane methodology recommendations were followed to guarantee the quality of the systematic review and statistical analysis [12,13] (Tables S1 and S2). The protocol was registered in PROSPERO (International Prospective Register of Systematic Reviews) (CRD42022329952) and followed without deviations.

The “PICO” (i.e., population, intervention, comparator, outcome) framework was applied to formulate the clinical question and define the qualifying requirements. The population included adult patients (age ≥ 18 years), diagnosed with MS, receiving adequate disease-modifying and antispastic therapy. The intervention was nabiximols as an add-on treatment. For primary outcomes, comparable parameters on MS-associated symptoms other than spasticity and pain were collected. These included self-reported parameters, such as the numerical rating scale (NRS) or visual analogue scale (VAS) for sleep disruption, bladder function, and depression-related parameters (primarily: Beck’s Depression Inventory). Secondary outcomes included the change in the subject global impression of change (SGIC); gait function, measured by the timed 10 m or 25 ft walk (assumed under the term: timed walk) and anxiety-related parameters.

In terms of study design, case reports, case series, cross-sectional questionnaires, non-peer reviewed articles, conference abstracts, and registers without corresponding peer-reviewed articles were excluded. Observational studies were included given the limitations and heterogeneity of the randomized controlled studies. In line with the exploratory nature of the present analysis, these studies were considered as relevant sources of information, as noted by Cheurfa et al. (2024) [14]. In terms of study design, case reports, case series, cross-sectional questionnaires, non-peer reviewed articles, conference abstracts, and registers without corresponding peer-reviewed articles were excluded. Observational studies were included given the limitations and heterogeneity of the randomized controlled studies. In line with the exploratory nature of the present analysis, these studies were considered as relevant sources of information, as noted by Cheurfa et al. (2024) [14].

The original literature search was conducted in 2022 in the Web of Science, MEDLINE (via PubMed), Cochrane Central Register of Controlled Trials (CENTRAL) and Embase databases. However, due to the elapsed time since the initial search, the literature search was updated on September 10, 2025. The following search key was used: ((tetrahydrocannabinol and cannabidiol) OR (THC and CBD) OR “thc/cbd” OR THC-CBD OR nabiximols OR sativex) and (multiple sclerosis), without any filters or restrictions.

### 2.2. Selection

Selection was conducted using Endnote 20 software (Clarivate Analytics, Philadelphia, PA, USA). Both automatic and manual duplicate removal were then performed by DK. Four independent investigators (DCS, DK, DG, and ILH) performed the selection in two stages: by title and abstract, and subsequently full-text contents. After each stage of the selection process, the inter-rater agreement was assessed using Cohen’s kappa coefficient (k), as described by McHugh (2012) [15]. Disagreements were resolved by a third independent investigator (NR) and by consensus at each step of the selection process. Retracted articles were checked, and corresponding records from trial registries were added when additional relevant data were available.

### 2.3. Data Collection and Writing

Data were extracted manually by DK, DG, and DM using a standardized Excel sheet (Microsoft Corporation, Redmond, Washington, USA) and were checked by another independent investigator (DG and DM for DK; DG and DK for DM; DK and DM for DG). The following data were collected from each eligible article: publication details [authors, year of publication, country of origin, digital object identifier (DOI)]; study characteristics (study type and study identifier, follow-up period, sample size); patient characteristics (sex distribution, age, disease type, spasticity features); posology of nabiximols (dose, route of administration and duration of treatment); information on outcomes (self-reported state of sleep disruption and bladder function; depression- and anxiety-related parameters; SGIC) as reported in each eligible article.

During data extraction, overlapping patient populations were avoided. In cases of repeated measures, all available data for meta-regression were collected. For conventional meta-analyses, short-term (<4 months) and long-term (last reported assessment after 4 months) outcomes were analyzed separately. In crossover studies, data reported in the peer-reviewed publication were prioritized. If these data were insufficient for analysis, supplementary information was retrieved from the corresponding clinical trial registry, where outcomes were sometimes reported separately for each study group.

Gait function was assessed by recalculating and pooling together the 10 m or 25 ft timed walk. These measures were considered comparable assessments of walking ability in clinical settings [16].

The quality and the number of spasms were assessed as two different features, as they were discussed separately in several articles. Spasm quality and spasm frequency were evaluated as separate outcomes because they were reported independently in several studies. Spasm quality reflects the subjective burden and severity of spasms experienced by patients and was assessed using different instruments, including Section 3 of the Multiple Sclerosis Spasticity Scale-88 (MSSS-88), a 0–3 spasm severity scale, 0–10 NRS, 0–10 VAS or 0–100 VAS. These measures were pooled because they all aimed to assess the perceived severity or impact of spasms despite differences in scoring systems.

In contrast, spasm frequency represented a more objective outcome and was consistently reported as the number of spasms over a defined period, including daily spasm counts, the average number of spasms per day during the previous week, or the number of spasms within the last 24 h. Therefore, these measures were considered sufficiently comparable to be pooled as indicators of spasm frequency.

When data were available only in graphical format, Plot Digitizer was used to convert them into numerical data. In cases of inconsistencies between the data reported in the published article and those reported in the protocol registry, data were extracted from the peer-reviewed publication [17].

### 2.4. Statistical Analysis

Initially, the before–after mean differences (MDs) were calculated for all studies in which the within-group mean change between time points was not recorded. For spasm quality, bladder function, and sleep-related outcomes, values were transformed to a common 0–100 scale using the original instrument range, taking the minimum scale value into account. The direction of effect was preserved during transformation, with changes interpreted according to the original scoring system of each instrument. No transformation was required for spasm frequency, as all studies reported comparable measures of daily spasm counts. For gait function, results from the 25-foot timed walk were converted to the equivalent 10 m timed walk, whereas data already reported as 10 m timed walk required no further adjustment. A correlation coefficient (*r*) of 0.6 was used to calculate the standard deviation of change. However, models with lower and higher correlation values were also run, obtaining numerically similar results (Table S3). In all cases, the baseline (time point 0) was used as the reference.

To assess the overall effect of treatment on outcomes, values were pooled using random-effects meta-analysis models with the inverse variance weighting methodology [18]. Where applicable, randomized controlled trials and observational studies were separated to potentially account for a proportion of the heterogeneity and to aid in clearer inference of model results.

For the global perception of change (SGIC), random-effects meta-analysis models were performed for odds ratios (ORs). The DerSimonian and Laird method was used to estimate the between-study variance (*τ*^2^) and the ORs were pooled using the inverse variance method [18].

To analyze the proportion of responders, a simple ratio meta-analysis was conducted. This involved pooling the proportions of responders across studies using random-effects models, and the overall proportion was calculated by weighting each study by the inverse of its variance.

All estimated confidence intervals were corrected using the Hartung–Knapp method [19]. To estimate the heterogeneity variance measure (*τ*^2^), the restricted maximum-likelihood (REML) estimator was used with the Q-profile method for the confidence interval [18]. Heterogeneity was assessed using the Higgins and Thompson *I*^2^ value [20]. Small study bias was assessed by visually inspecting funnel plots [21].

To evaluate the temporal effect on treatment outcomes, meta-regressions were fitted for all outcomes where the covariate was time of measurement. In these meta-regressions, the time variable was coded as a continuous variable representing the number of weeks (or months) post-intervention. Temporal meta-regressions (MD~Timepoint) were conducted using all available data, acknowledging that treatment discontinuation and open-label extension designs may contribute to residual heterogeneity.

All analyses were performed using R version 4.2.1 with the following packages: dmetar [22], metafor [18], and estmeansd [23].

Due to the exploratory design of this review, no formal sample-size calculation was applied, and this limitation was noted in the Discussion.

### 2.5. Risk of Bias Assessment and Certainty of Evidence Assessment

The assessment of bias factors was performed by two independent investigators (DK and DG). The Cochrane risk of bias tool (RoB2) was used to compare SGIC between placebo and nabiximols groups [24]. Other parameters were assessed using the developed version of the Risk Of Bias In Non-randomized Studies of Interventions, Version 2 (ROBINS-I V2) tool (https://www.riskofbias.info/welcome/robins-i-v2 (accessed on 10 June 2026) [25]. For visualization, the robvis tool was used [26].

The certainty in the body of evidence was evaluated using the Grading of Recommendations, Assessment, Development and Evaluations (GRADE) framework (DK, checked by DG) [27].

## 3. Results

### 3.1. Study and Patient Characteristics

**Figure 1:**
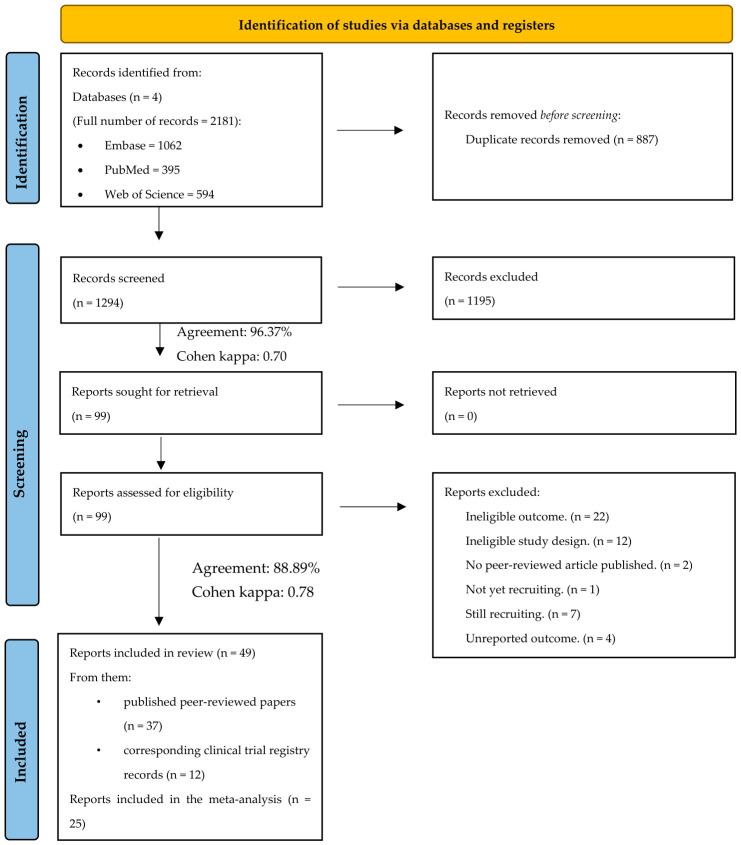
Flow diagram of study identification and selection based on PRISMA 2020 guidelines [12].

A total of 49 eligible articles were identified for systematic review, including the registered trial protocols for 12 of the studies. Of these, 25 articles (2949 patients) were included in the meta-analysis. The selection process is detailed in Figure 1.

The mean duration of treatment varied between 3 weeks and 72 weeks [28,29]. Detailed information can be found in Table S4a–d. The mean age of the patient groups ranged from 42 (± 8.9) years to 56.1 (± 8.6) years [30,31]. Prior to study enrollment, the shortest duration of MS was a median of 7.58 years (range: 0.3–23.0), as reported in the study by Maniscalco et al. (2018) [31]; the longest duration, as reported in the study by Mallada Frechín et al. (2018), was a mean of 24.6 (± 22.9) years [32]. The proportion of females in the nabiximols-treated groups ranged from 44% to 82% [33,34]. As nabiximols is primarily administered to relieve spasticity, baseline spasticity NRS was also collected, ranging from 3.87 to 8.7 [35,36]. The selected studies included a wide range of disease phenotypes. Although the progressive form was generally more common, the relapsing–remitting form was even more prevalent (77%) in the study by Paul and Silván (2021) [37]. Detailed patient characteristics can be found in Table S5a–e. Twenty-five studies were included in the meta-analysis [29,31,32,33,34,35,36,37,38,39,40,41,42,43,44,45,46,47,48,49,50,51,52,53,54]. All other papers were only assessed in the qualitative analysis [28,55,56,57,58,59,60,61,62,63,64,65].

### 3.2. Meta-Analysis of Clinical Data on the Efficacy of Nabiximols

**Figure 2:**
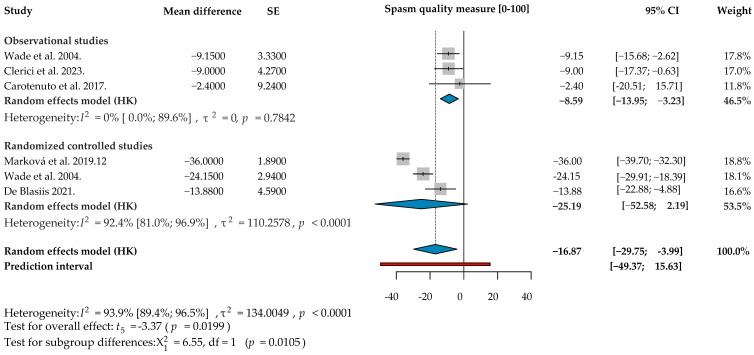
Effects of nabiximols on spasm quality. The meta-analysis of studies shows a statistically significant effect of nabiximols on spasm quality (*p* = 0.01). (SE: standard error) [38,41,46,49,50].

**Figure 3:**
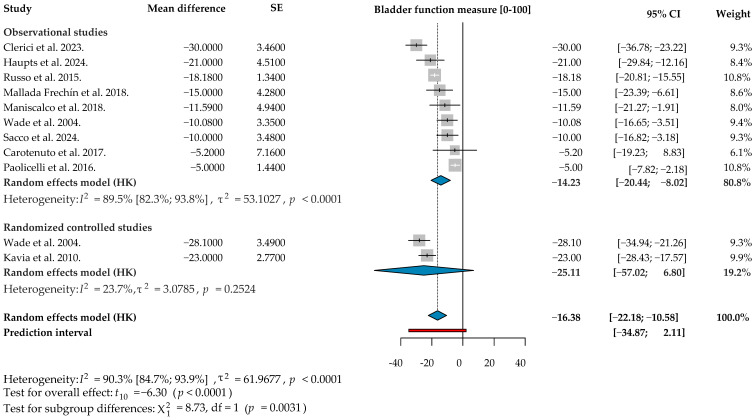
Effects of nabiximols on bladder function. The meta-analysis of studies shows a statistically significant effect of nabiximols on bladder function (*p* < 0.01). (SE: standard error; CI: confidence interval) [31,32,36,38,44,49,50,51,52,54].

**Figure 4:**
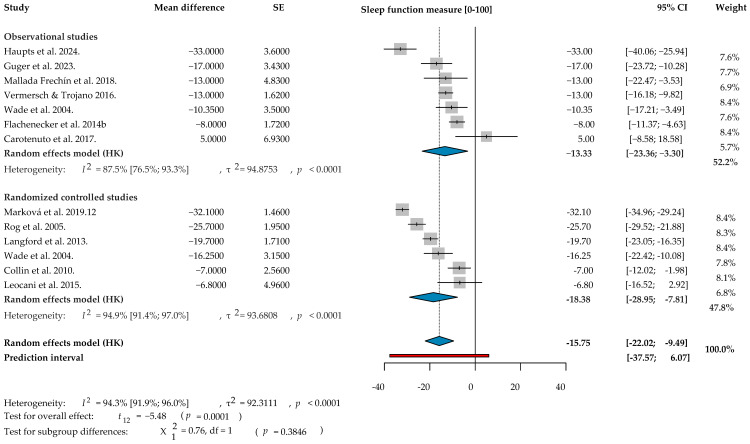
Effects of nabiximols on sleep disruption. The meta-analysis of studies shows a statistically significant effect of nabiximols on sleep disruption (*p* < 0.01). (SE: standard error; CI: confidence interval) [32,33,34,38,40,43,45,46,48,49,51,53].

**Figure 5:**
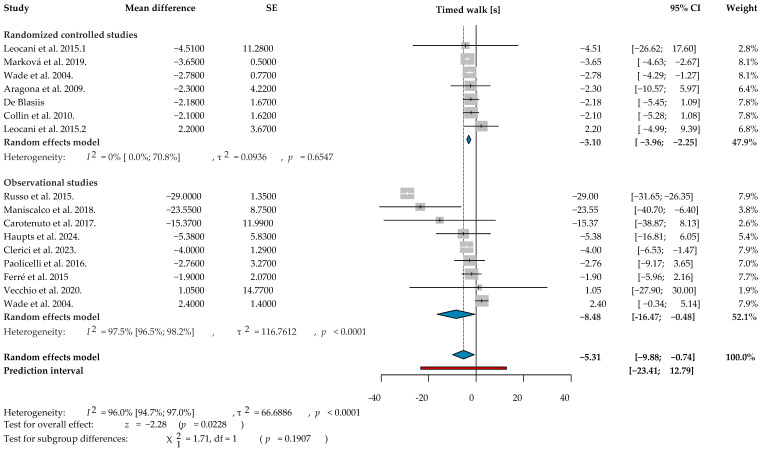
Effect of nabiximols on gait function (measured in timed walk (s)). The meta-analysis of the studies shows a statistically significant effect of nabiximols on gait function, measured by a 10 m or 25 ft timed walk (*p* = 0.03). (SE: standard error; CI: confidence interval) [29,31,33,36,38,40,41,42,46,47,49,50,51,54].

**Figure 6:**
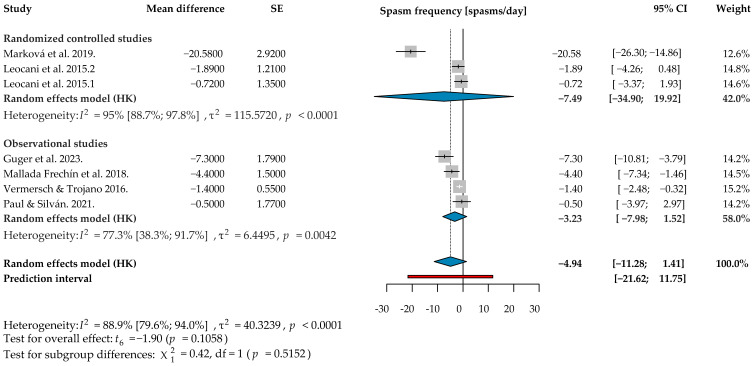
Effects of nabiximols on spasm frequency. The meta-analysis of the studies shows a statistically non-significant effect of nabiximols on the frequency (*p* = 0.09). (SE: standard error; CI: confidence interval) [32,33,37,46,48,53].

The primary objective of this study was to evaluate the efficacy of nabiximols in alleviating symptoms associated with MS beyond spasticity and pain and to ascertain whether any observed effect had therapeutic value. For this reason, a cross-temporal meta-regression was conducted to determine whether the effects were merely transient or whether using nabiximols as prescribed would be a viable option for relieving several symptoms. The published literature supported a temporal meta-regression for bladder function, sleep disruption, spasm quality, spasm frequency, and gait function. In addition, a non-temporal meta-analysis was also conducted for these cases (Figure 2, Figure 3, Figure 4, Figure 5 and Figure 6; Figures S1–S5).

According to the results, the literature does not currently provide sufficient evidence to confirm a significant time dependence of efficacy in the case of bladder function, gait function, sleep disruption, spasm quality, and spasm frequency (Figures S1 to S4; Table 1). However, it should also be noted that symptom-worsening was also not proven.

On the other hand, statistically significant effects were observed in the non-temporal meta-analysis for spasm quality (MD = −16.87; 95% CI = (−29.75)–(−3.99); I^2^ = 94%; Figure 2), bladder function (MD = −16.38; 95% CI = (−22.18)–(−10.58); I^2^ = 90%; Figure 3), amelioration of sleep disruption (MD = −15.75; 95% CI = (−22.02)–(−9.49); I^2^ = 94%; Figure 4) and in gait function (timed walk MD (s) = −5.31; 95% CI (s) = (−9.88)–(−0.74); I^2^ = 96%; Figure 5). These changes were also observed in other publications that could not be included in the statistical analysis due to inappropriate reporting (i.e., using different types of measures). For this reason, these studies will be discussed in greater detail in the subsequent systematic review.

In contrast, the meta-analysis did not demonstrate a significant difference in spasm frequency (spasm frequency MD = −4.94; 95% CI = (−11.28)–(1.41); I^2^ = 89%; Figure 6). However, most studies in this case also point in one direction and nabiximols is generally an effective agent.

**Figure 7:**
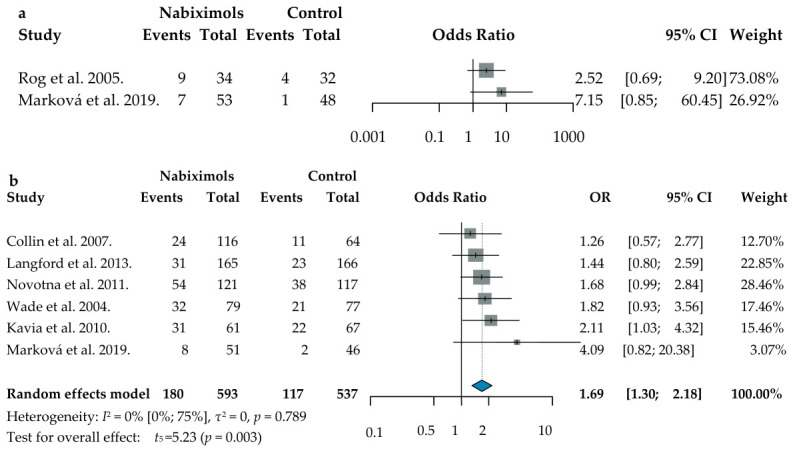
Comparison of the effects of nabiximols and a control drug on the subject global perception of change (SGIC) for subjects with multiple sclerosis (MS). (OR: odds ratio; CI: confidence interval). (**a**) The analysis of short-term results is not feasible due to the small number of studies. (**b**) Meta-analysis of long-term studies (>1 month) shows a statistically significant effect (*p* < 0.01) of nabiximols compared to a control group on the overall dichotomized measure, SGIC [34,35,39,44,45,46,49].

**Figure 8:**
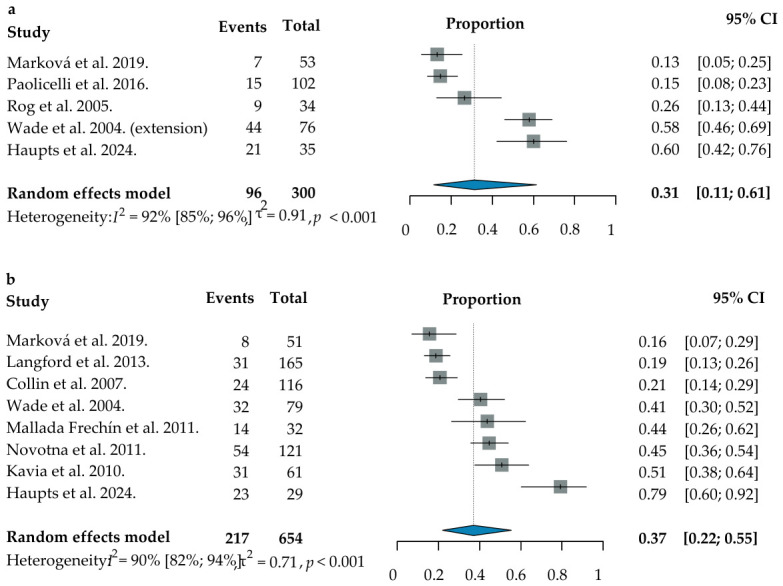
Nabiximols efficacy measured by subject global perception of change (SGIC). According to the meta-analysis, the proportion of responders by SGIC within a month (**a**) is 31%, while after a month (**b**) it reaches 37%. (SE: standard error; CI: confidence interval) [32,34,35,36,39,44,45,46,49,51].

Overall, the meta-analysis suggested that nabiximols may exert beneficial effects across multiple symptom domains. Meta-regression analyses on the other hand did not identify a statistically significant association between treatment duration and treatment effect. However, SGIC should be highlighted (Figure 7 and Figure 8) as a parameter that can be used as an overall measure. While comparisons with the controls are not feasible for less than one month (Figure 7a), long-term therapy showed a significantly better performance (responders by SGIC = 1.69; 95% CI = (1.30)–(2.18); I^2^ = 0%; Figure 7b). If only the proportion of responders is evaluated, it is noteworthy that a slight increase (6%) can be noticed after the first month of treatment (proportion of responders by SGIC = 0.31; 95% CI = (0.11)–(0.61); I^2^ = 92%; Figure 8a vs. proportion of responders by SGIC = 0.37; 95% CI = (0.22)–(0.55); I^2^ = 90%; Figure 8b).

### 3.3. Qualitative Analysis of the Efficacy of Nabiximols

Due to research features and data characteristics, appropriate statistical analysis was not feasible for anxiety and depression. As a result, studies were collected that provided precise data on the effectiveness of nabiximols compared to pretreatment situations.

Depression- and general mental health-related outcomes in both short- and long-term settings mainly showed a favorable trend (Table S6) [29,55]. The available studies used heterogeneous instruments, including measures of depression, mood, and general mental health. While most studies reported improvements after nabiximols treatment, the diversity of outcome measures did not allow firm conclusions specifically regarding depression.

For anxiety-related parameters, it was difficult to draw any conclusions due to conflicting data (Table S7).

The overall evidence showed only a few negative changes, and in most cases, moderate or significant improvements were seen in bladder function, sleep disruption, spasm quality, spasm frequency, gait function (timed walk) and depression-related parameters. As an overall parameter, SGIC also indicated improvements. Furthermore, auxiliary results underline this broadly positive statement, a good example being the gait function assessed by the 10 m or 10 ft timed walk. However, as Coghe et al. (2015) employed alternative types of measurement, it was impossible to pool with the timed walk; nevertheless, they found that nabiximols improved gait features [57].

### 3.4. Bias Analysis and Evidence Level by Grading of Recommendations, Assessment, Development and Evaluations (GRADE)

RoB2 analyses were performed for the comparative SGIC cases. With the exception of one instance (Marková et al. 2019, week 12 [46]), the overall results were “low risk” or “some concerns”. This notable case involved a significant number of missing participants when data were traced back from the trial registry website (Table S8).

For other parameters analyzed by ROBINS-I, a generally serious overall risk of bias was observed (Figure S6a–i). The most common sources of bias were selection bias (domain 2) and bias due to missing data (domain 5). Across the whole study, these were classified as “serious” in 44 and 41 cases, respectively. On the other hand, selective reporting appears to be negligible.

In general, low quality of evidence should be considered for each measure (Tables S9 and S10). This is generally determined by heterogeneous study designs and measurement methods. It should be noted that only a few articles were available for evaluation, and some studies were still in the pilot stage, resulting in a small patient sample size.

## 4. Discussion

The aim of our study was to systematically review and meta-analyze the effects of nabiximols on MS-associated symptoms other than spasticity. The statistical analysis of 25 studies suggests possible signals of benefit for bladder function, sleep disruption, spasm quality, and gait function in MS. Given the substantial heterogeneity across studies, these findings should not be regarded as firm evidence of broad symptomatic efficacy and require confirmation in future studies. Furthermore, the percentage of SGIC responders slightly increased after the first month (GRADE: very low). These findings raise the question of whether the conventional 4-week trial period is sufficient for all patients [66]. Our results suggest that a month-long trial may not be sufficient for all individuals and that patients may benefit from alleviation of symptoms other than spasticity; however, as the high heterogeneity indicates, this analysis must be accepted only in the context of an exploratory study (Table 2).

These results highlight that nabiximols may be effective in treating several symptoms associated with MS. Although it seems clear that tetrahydrocannabinol acts on cannabinoid receptors, the role of cannabidiol is more complex [4]. Previous studies have shown that cannabidiol reduces the euphoric effect of tetrahydrocannabinol and enhances its anti-spasticity effect [67]. Furthermore, cannabidiol binds to more than 20 receptors, including transient receptor potential vanilloid (TRPV) channels and serotonin receptors, and influences several liver enzymes, underlying its diverse pharmacological profile. Castillo-Arellano et al. (2023) report that cannabidiol may be a promising anxiolytic, antidepressant, antinociceptive, antiaddictive, and anti-inflammatory agent, whose effects may also be relevant for MS patients [68].

Notably, Fernández et al. (2020) rather focus on the MS-related damage across multiple regions of the central nervous system [4]. They hypothesize a so-called “spasticity-plus syndrome”, referring to a cluster of interrelated symptoms mediated (at least partly) through overlapping brainstem areas. Therefore, a well-targeted therapy may mitigate not only spasticity but also accompanying symptoms. As this area appears to have an abundance of cannabinoid receptors, nabiximols is potentially an appropriate choice. These considerations are supported by our results and are also reflected in a recently proposed Italian treatment algorithm, published by Annovazzi et al. (2026) [7].

In addition, qualitative analysis of clinical data showed promising results in both short- and long-term assessments of depression and general mental health-related parameters; however, no notable effects were found in anxiety-related parameters. These results are in agreement with recent publications. Bilbao and Spanagel (2022) also found a significant effect on sleep disruption, but no efficacy for the so-called “symptoms associated with MS” (a non-specified parameter) and anxiety [69]. However, the authors further extended the scope of their analysis to cannabis products other than nabiximols. Our previous meta-analysis also supported the theory that nabiximols may be useful in treating sleep disruption, improving gait function, and improving SGIC [8].

### 4.1. Strengths and Limitations

The most important strength of our study is that the statistical analysis utilized the most comprehensive source information accessible from patients with MS, including both randomized and non-randomized studies. This, combined with a detailed and thorough approach, means that the data produced in this study are among the most accurate currently available.

One limitation of our study is that the meta-analyses are primarily based on secondary outcomes of clinical trials, and therefore the sample sizes were often not disclosed. Due to the exploratory nature of our study, we did not perform a formal sample-size calculation. Furthermore, while randomized controlled trials provide the highest level of evidence, a substantial proportion of the available literature consists of open-label and before–after studies. These studies were included because they provide valuable information regarding symptoms other than spasticity, which was the primary focus of the present analysis. However, uncontrolled study designs are inherently limited, as they are susceptible to placebo effects, regression to the mean, and natural fluctuations in symptom severity over the course of multiple sclerosis. Consequently, observed improvements may partly reflect the underlying disease course rather than a true treatment effect.

In addition, differences in outcome definitions and assessment methods required mathematical recalculations for most analyses, with the exception of daily spasm counts. This is especially true for gait function. It was assessed using different short-distance walk tests (10 m and 25 ft walk), which were pooled based on their common assessment of walking ability. Nevertheless, differences in test distance may have contributed to heterogeneity. Furthermore, the varying measures prevented the statistical analysis of data on anxiety and depression. Although the broad spectrum of study types allowed us to provide more information on the effects of nabiximols, it is also a limitation, as pooling data from different study designs may introduce bias. Therefore, the results should be interpreted with caution, as substantial clinical heterogeneity existed among the included studies despite the statistical assessment of heterogeneity. Our risk of bias analysis also revealed serious concerns, for which we must highlight two main sources of bias. First, nabiximols is effective only in a limited number of patients; according to the summary of product characteristics, treatment should be discontinued if no relevant improvement is seen within a month. This mainly affects domain 2 of the ROBINS-I tool. The second source of bias to be highlighted is missing data (domain 5), which is mostly related to the observational design of the included studies and, in some cases, to the exclusion of previously mentioned non-responders.

### 4.2. Implications for Clinical Practice and Research

Translational medicine aims to support the implementation of scientific evidence into clinical practice [70,71]. In this context, nabiximols has the potential to play a role in treating patients with multiple sclerosis presenting with several concurrent symptoms when first-line symptomatic therapies are insufficient, in accordance with the “spasticity-plus syndrome” concept. These findings, based on low to very low certainty evidence, should be interpreted as exploratory signals rather than evidence of clinical effectiveness. Any potential implications regarding polypharmacy remain hypothesis-generating and warrant further investigation in studies with systematic assessment of safety and tolerability [72,73].

## 5. Conclusions

This work represents an exploratory analysis and the results should be interpreted with caution. The findings suggest a potential benefit of nabiximols on sleep disruption, bladder function, spasm quality, and gait function; however, the low certainty and high heterogeneity of the included studies limit the strength of these conclusions. Furthermore, the SGIC results may indicate a delayed response in some patients. In this context, particularly for outcomes beyond spasticity, the findings raise the question of whether the standard trial period is sufficient for all patients, while acknowledging the overall low certainty of the evidence.

## Figures and Tables

**Table 1:** Statistical values of cross-temporal meta-regressions. (Tau^2^: estimated amount of residual heterogeneity; S.E.: standard error; β: regression coefficient).

Measure	Tau^2^	S.E.	β	*p*
Bladder function	69.2	39.2	0.227	0.7741
Gait function (timed walk (s))	70.9	33.4	0.253	0.6464
Sleep disruption	97.7	46.4	−0.465	0.5692
Spasm quality	162.1	129.8	−0.470	0.7677
Spasm frequency	42.8	28.9	−0.645	0.4158

**Table 2:** Summary of findings. Risk of bias was assessed by Risk Of Bias In Non-randomized Studies of Interventions (ROBINS-I) tool, and certainty of evidence was assessed by Grading of Recommendations, Assessment, Development and Evaluations (GRADE) framework. (CI: confidence interval; MD: mean difference).

Outcome		MD	95% CI	Risk of Bias	Certainty of Evidence	Comment
Spasm quality		−16.87	(−29.75)–(−3.99)	Serious	Very low	No confirmed time-dependency
Bladder function		−16.38	(−22.18)–(−10.58)	Serious	Very low	No confirmed time-dependency
Sleep disruption		−15.75	(−22.02)–(−9.49)	Serious	Very low	No confirmed time-dependency
Gait function (timed walk (s))		−5.31	(−9.88)–(−0.74)	Serious	Very low	No confirmed time-dependency
Spasm frequency		−4.94	(−11.28)–(1.41)	Serious	Very low	No confirmed time-dependency
**Outcome**		**Proportion**	**95% CI**	**Risk of Bias**	**Certainty of Evidence**	**Comment**
SGIC	<1 month	0.31	0.11–0.61	Serious	Very low	
	>1 month	0.37	0.22–0.55	Serious	Very low	Nabiximols showed significant improvement vs. control (OR = 1.69; 95% CI: 1.30–2.18)
**Outcomes (no statistical analysis)**			**Qualitative Analysis**	**Risk of Bias**	**Certainty of Evidence**	**Comment**
Depression and general mental health-related outcomes			Improvement can be seen in general	Serious	Very low	
Anxiety-related parameters			Conflicting results	Serious	Very low	

## Data Availability

The original contributions presented in this study are included in the article/Supplementary Materials. Further inquiries can be directed to the corresponding author.

## Supplementary Materials

The following supporting information can be downloaded at: https://www.mdpi.com/article/10.3390/medsci14030346/s1, Figure S1. Effects of nabiximols on spasm quality between the 4th and 12th week. The temporal meta-analysis shows the change in the severity of spasm quality over time. Relevant time dependence on the relief of bladder disfunction was not found after four months (p = 0.5492). The points in the graph show the studies utilised, and the size of the points shows the weight of the study; Figure S2. The effects of nabiximols on bladder function between the 4th and 12th week. The temporal meta-analysis shows the change in the severity of bladder dysfunction over time. Relevant time dependence on the relief of bladder disfunction was not found after four months (p = 0.8184). The points in the graph show the studies utilised, and the size of the points shows the weight of the study; Figure S3. Effect of nabiximols on gait impairment measured in 10 m or 25 ft walk. The temporal meta-analysis shows the change in the gait-related 10 m or 25 ft timed walk in time. No time dependence has been found (p= 0.7203). The points in the graph show the studies utilized, and the size of the points shows the weight of the study; Figure S4. Effects of nabiximols on sleep disruption between the 4th and 12th week. The temporal meta-analysis shows the change in severity of sleep disruption over time. The relevant time dependence on alleviation of sleep disturbance was not found after four months of treatment (p = 0.3382). The points in the graph show the studies utilised, and the size of the points shows the weight of the study; Figure S5. Effects of nabiximols on spasm frequency between the 4th and 12th week. The temporal meta-analysis shows the change in the frequency of spasms in time. The relevant time dependence on the relief of spasm frequency was not found after four months of treatment (p = 0.3907). The points in the graph show the studies utilized, and the size of the points shows the weight of the study; Figure S6. Risk of bias assessment in the collected papers for bladder function (a); sleep disruption (b); spasm quality (c); spasm frequency (d); gait function in timed walk (e); feelings about the treatment (less than a month—f); feelings about the treatment (more than a month—g); depression-related parameters (h) and anxiety-related parameter (i). The ROBINS-I tool was used. Visualization was made with robvis tool; Table S1. PRISMA checklist for abstracts; Table S2. PRISMA checklist; Table S3. Models with lower and higher correlation values for statistical analysis; Table S4. Characteristics of studies included; Table S5. Patient characteristics; Table S6. Efficacy of nabiximols treatment on depression and general mental health parameters. Efficacy is separated by its length; cut-off point is one month. Data relates to the longest follow-up in all cases of long-term therapy; Table S7. Efficacy of nabiximols treatment on anxiety-related parameters. The data in the table are from research that provide exact details on parameters associated to anxiety. Efficacy is separated by its length, cut-off point is one month. Data relates to the longest follow-up in all cases of long-term therapy; Table S8. Risk of bias assessment of subjects’ global impression of change in long-term and short-term cases. The revised Cochrane risk-of-bias tool (RoB2) was used. The assessed features: D1: Randomisation process; D2: Deviations from the intended interventions; D3: Missing outcome data; D4: Measurement of the outcome; D5: Selection of the reported result; Table S9. Evaluation of evidence level of comparative measures by Grading of Recommendations, Assessment, Development and Evaluations (GRADE) framework; Table S10. Evaluation of evidence level of prognostic measures by Grading of Recommendations, Assessment, Development and Evaluations (GRADE) framework.

## Abbreviations

The following abbreviations are used in this manuscript:

CENTRAL	Central Register of Controlled Trials
CI	confidence interval
DOI	digital object identifier
GRADE	Grading of Recommendations, Assessment, Development and Evaluations
MS	multiple sclerosis
NRS	numerical rating scale
OR	odds ratio
PRISMA	Preferred Reporting Items for Systematic Reviews and Meta-Analyses
RoB2	Risk of bias was assessed by Cochrane risk of bias instrument
ROBINS-I	Risk Of Bias In Non-randomized Studies of Interventions
SGIC	subject global impression of change
TRPV	transient receptor potential vanilloid
VAS	visual analogue scale
