# Supplementary material for: Nabiximols in Multiple Sclerosis: Beyond Spasticity—An Exploratory Systematic Review and Meta-Analysis of Symptomatic Outcomes
Source: Med Sci (Basel). 2026 Jun 25;14(3):346. doi: 10.3390/medsci14030346 (PMC13413933; doi:10.3390/medsci14030346)
Supplement: Supplementary file 1 [file medsci-14-00346-s001.zip › Kleiner_2026.06.24_Supplementary.pdf]

Systematic Review

Nabiximols in Multiple Sclerosis: Beyond Spasticity—An Exploratory Systematic Review and Meta-Analysis of Symptomatic Outcomes

Dénes Kleiner [1,2,*], István László Horváth [1,2], Dóra Mátis [2], Rita Nagy [2,3], Dorottya Gergő [2,4], Katalin Lugosi [2,5], Gábor Fazekas [6,7], Péter Fehérvári [2,8], Péter Hegyi [2,9,10] and Dezső Csupor [2,9,11]

1 University Pharmacy, Department of Pharmacy Administration, Semmelweis University, Hőgyes Endre utca 7-9., 1092 Budapest, Hungary; horvath.istvan@semmelweis.hu
2 Centre for Translational Medicine, Semmelweis University, Baross utca 22, 1085 Budapest, Hungary; dora.matis@icloud.com (D.M.); nagyrita003@gmail.com (R.N.); gergo.dorottya@gmail.com (D.G.); lugosikacci@gmail.com (K.L.); fehervari.peter.biomat@gmail.com (P.F.); hegyi2009@gmail.com (P.H.); csupor.dezso@gmail.com (D.C.)
3 Heim Pál National Pediatric Institute, 1089 Budapest, Hungary
4 Department of Pharmacognosy, Semmelweis University, Üllői út 26., 1085 Budapest, Hungary
5 Multiple Sclerosis Centre, Bajcsy-Zsilinszky Hospital, Maglódi út 89-91, 1106 Budapest, Hungary
6 Rehabilitation Clinic, Semmelweis University, Szanatórium utca 19, 1121 Budapest, Hungary; fazekas.gabor@semmelweis.hu
7 Department of Rehabilitation, University of Szeged, Tisza Lajos körút 97, 6722 Szeged, Hungary
8 Budapest Department of Biostatistics, University of Veterinary Medicine, István utca 2., 1078 Budapest, Hungary
9 Institute for Translational Medicine, Medical School, University of Pécs, Szigeti út 12, 7624 Pécs, Hungary
10 Institute of Pancreatic Diseases, Semmelweis University, Tömő utca 25-29, 1083 Budapest, Hungary
11 Institute of Clinical Pharmacy, University of Szeged, Szikra utca 8, 6725 Szeged, Hungary
* Correspondence: kleiner.denes@semmelweis.hu; Tel.: +36-14591500

Supplementary Figures

Academic Editor: Umberto Aguglia

Received: 19 May 2026
Revised: 15 June 2026
Accepted: 23 June 2026
Published: 25 June 2026

Copyright: © 2026 by the authors. Submitted for possible open access publication under the terms and conditions of the Creative Commons Attribution (CC BY) license.

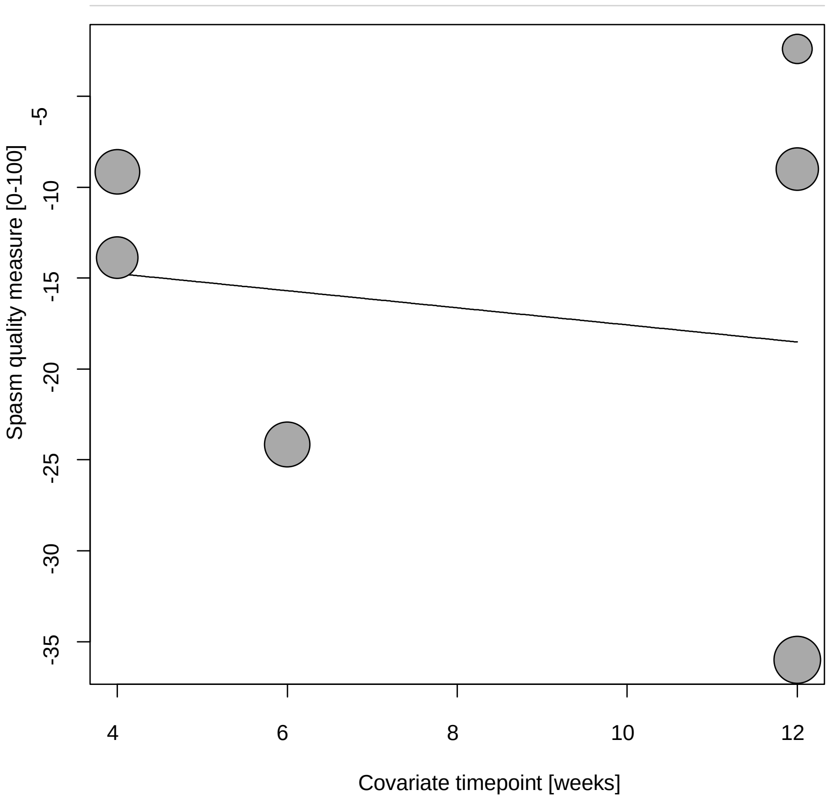

Figure S1

Figure S1. Effects of nabiximols on spasm quality between the 4th and 12th week. The temporal meta-analysis shows the change in the severity of spasm quality over time. Relevant time dependence on the relief of bladder disfunction was not found after four months (p= 0.5492). The points in the graph show the studies utilised, and the size of the points shows the weight of the study.

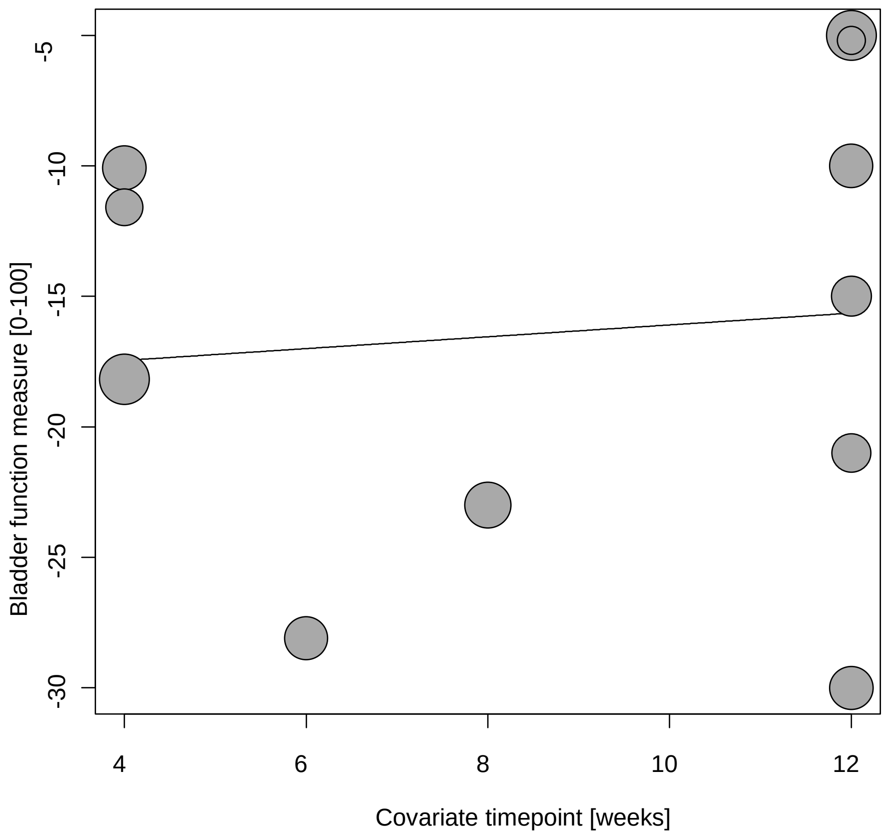

Figure S2

Figure S2. The effects of nabiximols on bladder function between the 4th and 12th week. The temporal meta-analysis shows the change in the severity of bladder dysfunction over time. Relevant time dependence on the relief of bladder disfunction was not found after four months (p= 0.8184). The points in the graph show the studies utilised, and the size of the points shows the weight of the study.

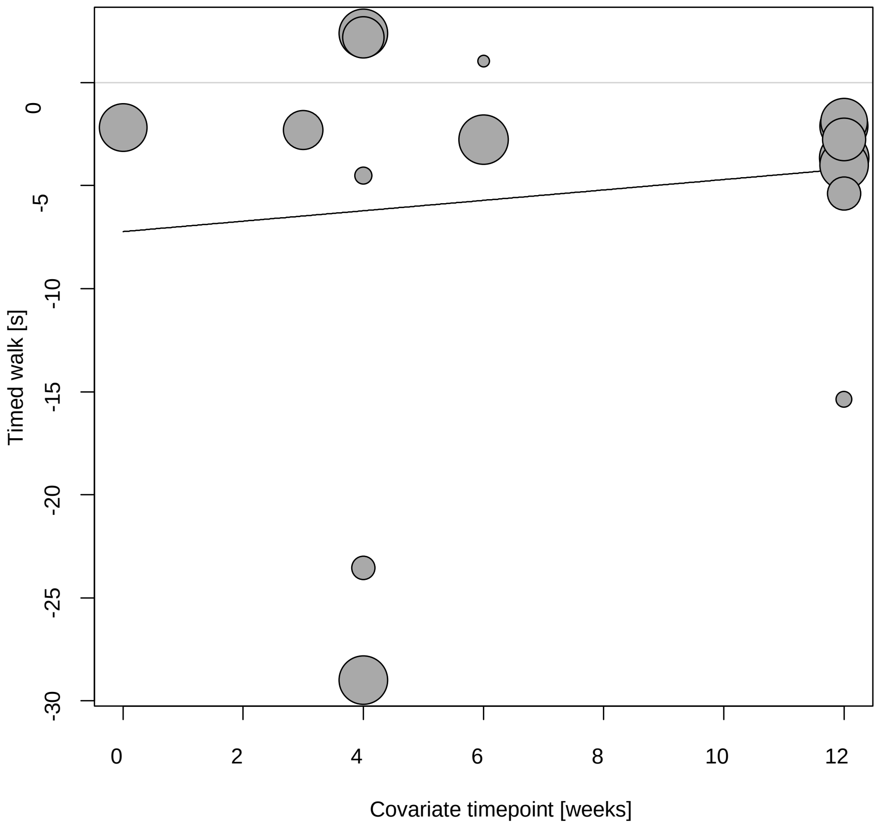

Figure S3

Figure S3. Effect of nabiximols on gait impairment measured in 10 m or 25 ft walk. The temporal meta-analysis shows the change in the gait-related 10 m or 25 ft timed walk in time. No time dependence has been found ($p = 0.7203$). The points in the graph show the studies utilized, and the size of the points shows the weight of the study.

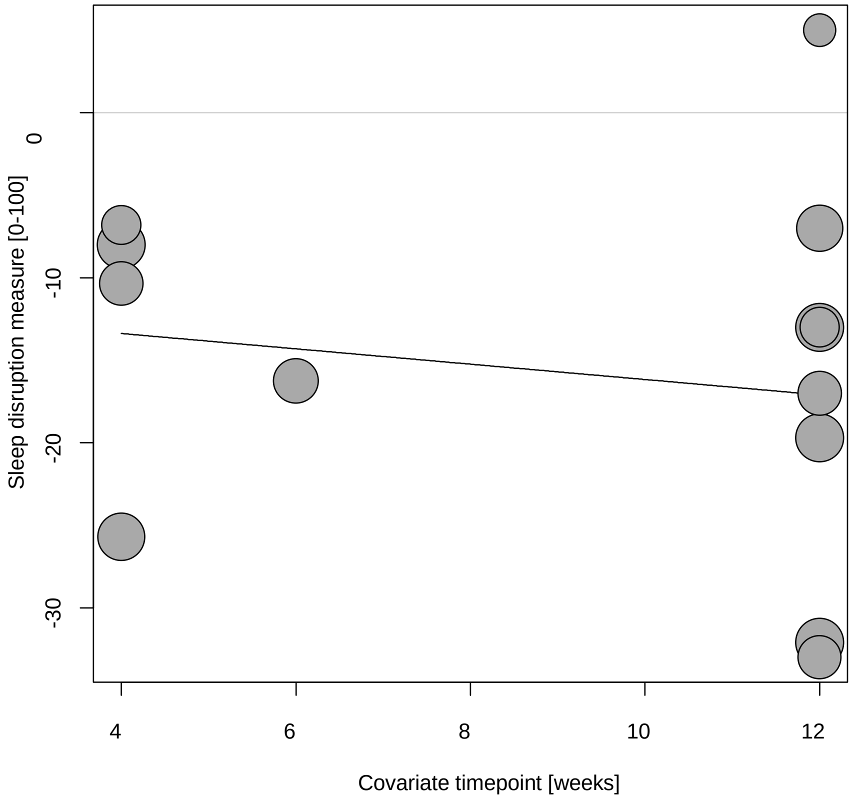

Figure S4

Figure S4. Effects of nabiximols on sleep disruption between the 4th and 12th week. The temporal meta-analysis shows the change in severity of sleep disruption over time. The relevant time dependence on alleviation of sleep disturbance was not found after four months of treatment (p= 0.3382). The points in the graph show the studies utilised, and the size of the points shows the weight of the study.

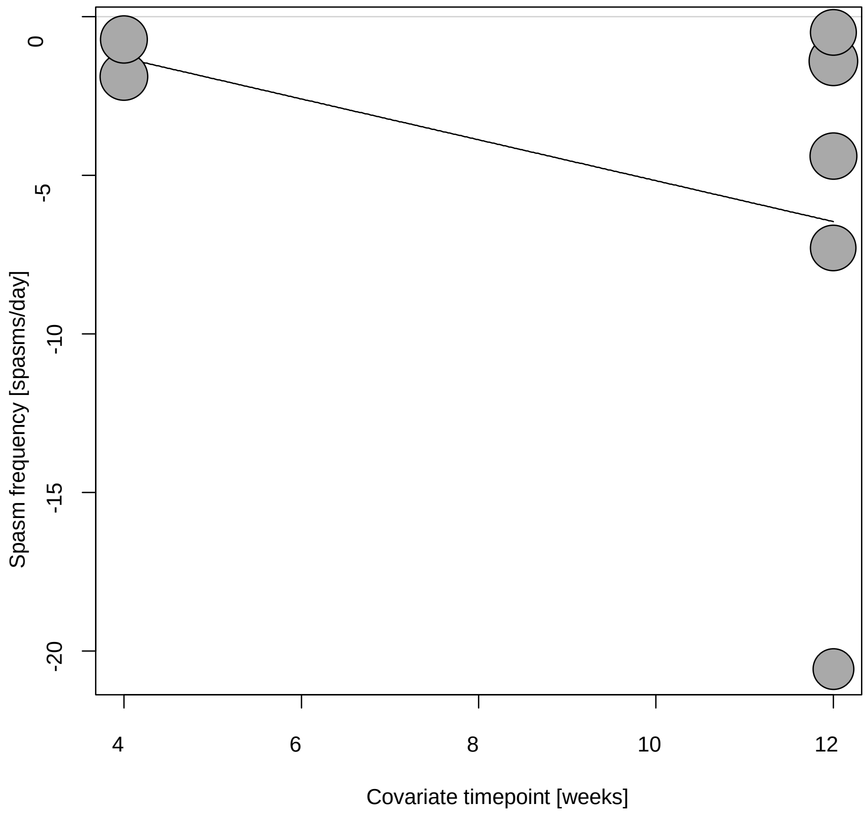

Figure S5

Figure S5. Effects of nabiximols on spasm frequency between the 4th and 12th week. The temporal meta-analysis shows the change in the frequency of spasms in time. The relevant time dependence on the relief of spasm frequency was not found after four months of treatment (p= 0.3907). The points in the graph show the studies utilized, and the size of the points shows the weight of the study.

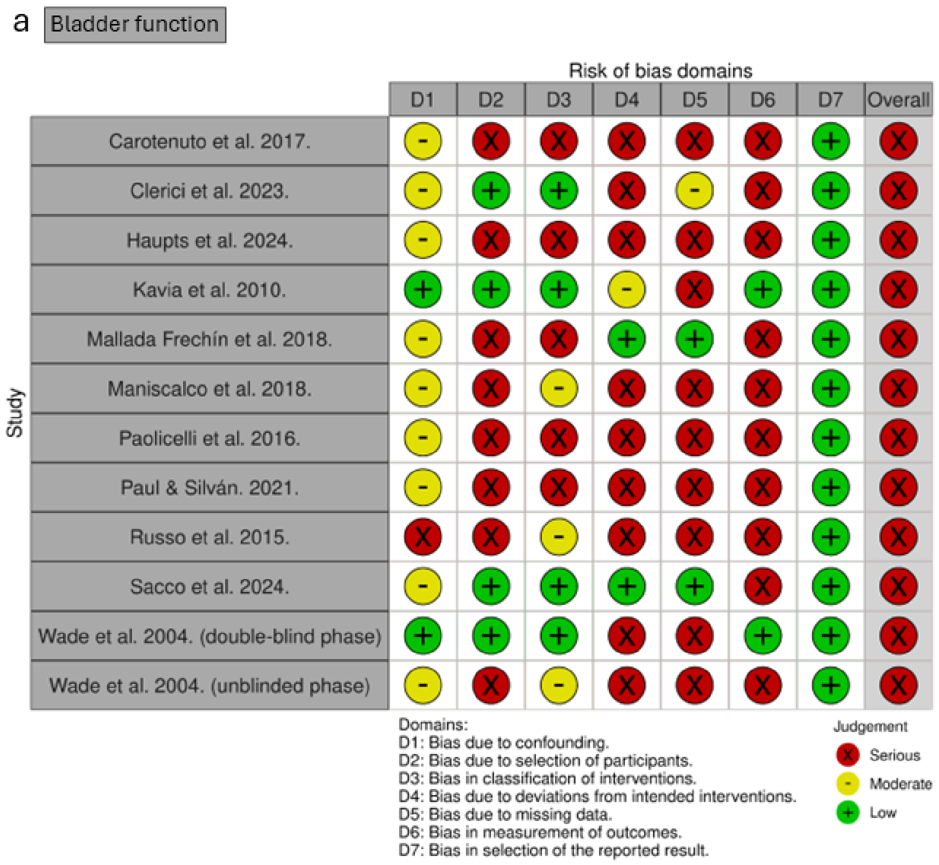
a
Bladder function
Risk of bias domains
D1
D2
D3
D4
D5
D6
D7
Overall
Study
Carotenuto et al. 2017.
Clerici et al. 2023.
Haupts et al. 2024.
Kavia et al. 2010.
Mallada Frechín et al. 2018.
Maniscalco et al. 2018.
Paolicelli et al. 2016.
Paul & Silván. 2021.
Russo et al. 2015.
Sacco et al. 2024.
Wade et al. 2004. (double-blind phase)
Wade et al. 2004. (unblinded phase)
Domains:
D1: Bias due to confounding.
D2: Bias due to selection of participants.
D3: Bias in classification of interventions.
D4: Bias due to deviations from intended interventions.
D5: Bias due to missing data.
D6: Bias in measurement of outcomes.
D7: Bias in selection of the reported result.
Judgement
Serious
Moderate
Low

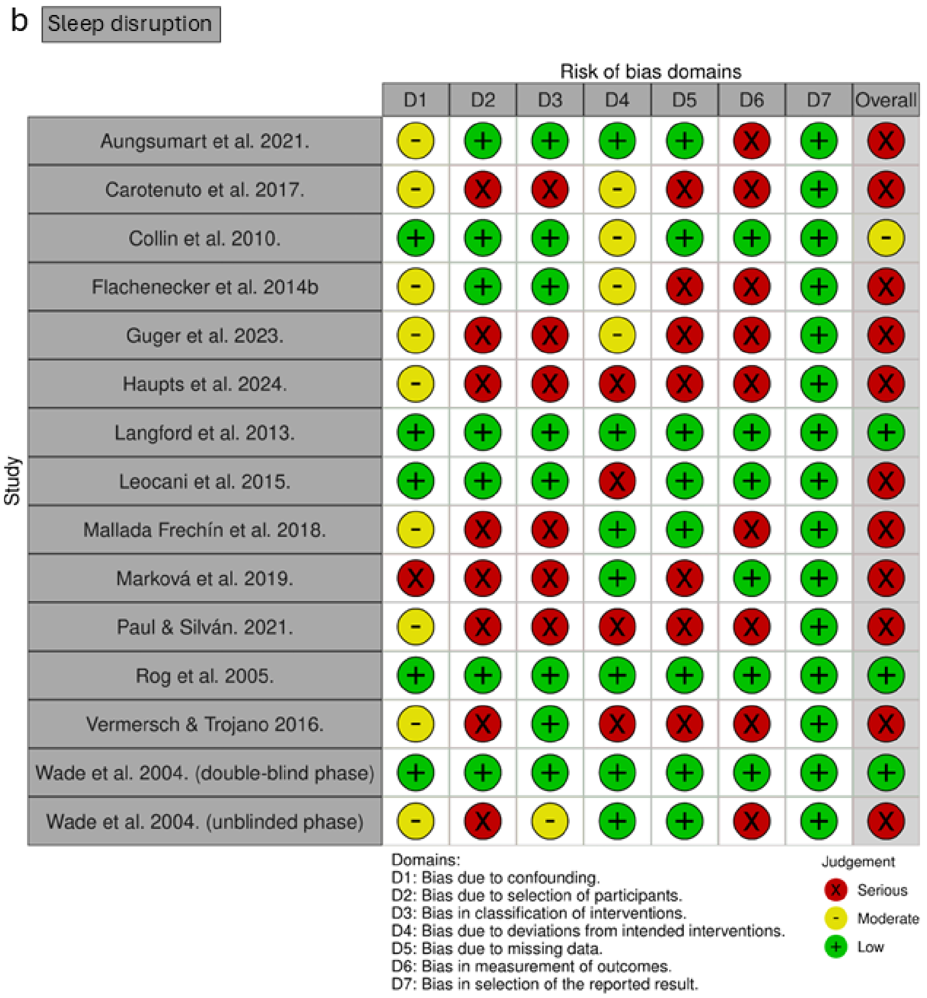
b
Sleep disruption
Risk of bias domains
D1
D2
D3
D4
D5
D6
D7
Overall
Study
Aungsumart et al. 2021.
Carotenuto et al. 2017.
Collin et al. 2010.
Flachenecker et al. 2014b
Guger et al. 2023.
Haupts et al. 2024.
Langford et al. 2013.
Leocani et al. 2015.
Mallada Frechín et al. 2018.
Marková et al. 2019.
Paul & Silván. 2021.
Rog et al. 2005.
Vermersch & Trojano 2016.
Wade et al. 2004. (double-blind phase)
Wade et al. 2004. (unblinded phase)
Domains:
D1: Bias due to confounding.
D2: Bias due to selection of participants.
D3: Bias in classification of interventions.
D4: Bias due to deviations from intended interventions.
D5: Bias due to missing data.
D6: Bias in measurement of outcomes.
D7: Bias in selection of the reported result.
Judgement
Serious
Moderate
Low

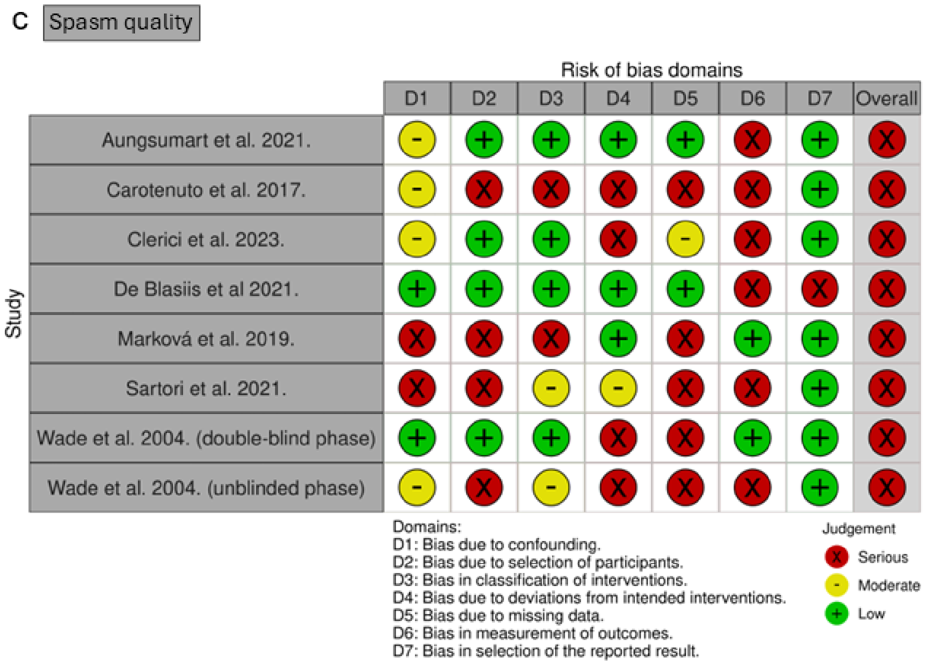
c
Spasm quality
Risk of bias domains
D1
D2
D3
D4
D5
D6
D7
Overall
Study
Aungsumart et al. 2021.
Carotenuto et al. 2017.
Clerici et al. 2023.
De Blasiis et al 2021.
Marková et al. 2019.
Sartori et al. 2021.
Wade et al. 2004. (double-blind phase)
Wade et al. 2004. (unblinded phase)
Domains:
D1: Bias due to confounding.
D2: Bias due to selection of participants.
D3: Bias in classification of interventions.
D4: Bias due to deviations from intended interventions.
D5: Bias due to missing data.
D6: Bias in measurement of outcomes.
D7: Bias in selection of the reported result.
Judgement
Serious
Moderate
Low

d Spasm frequency

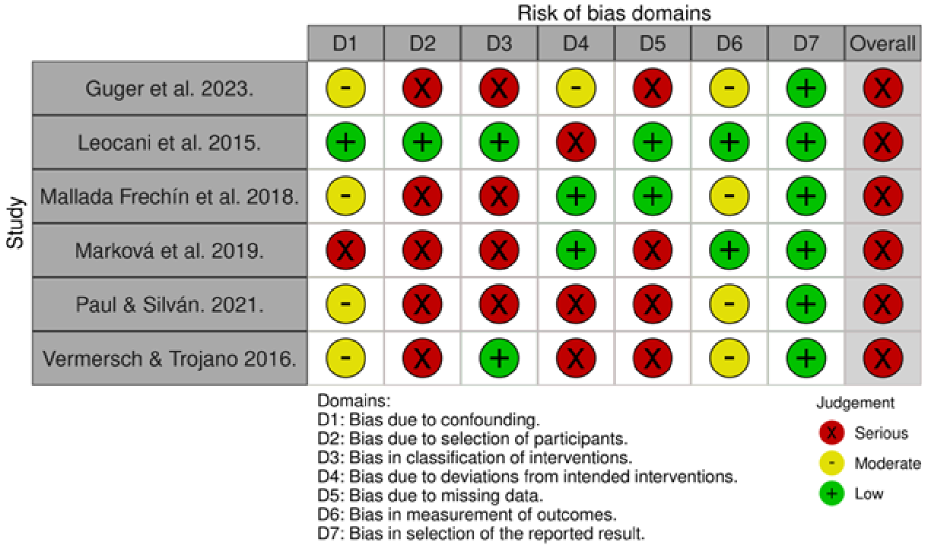

e Gait (timed walk)

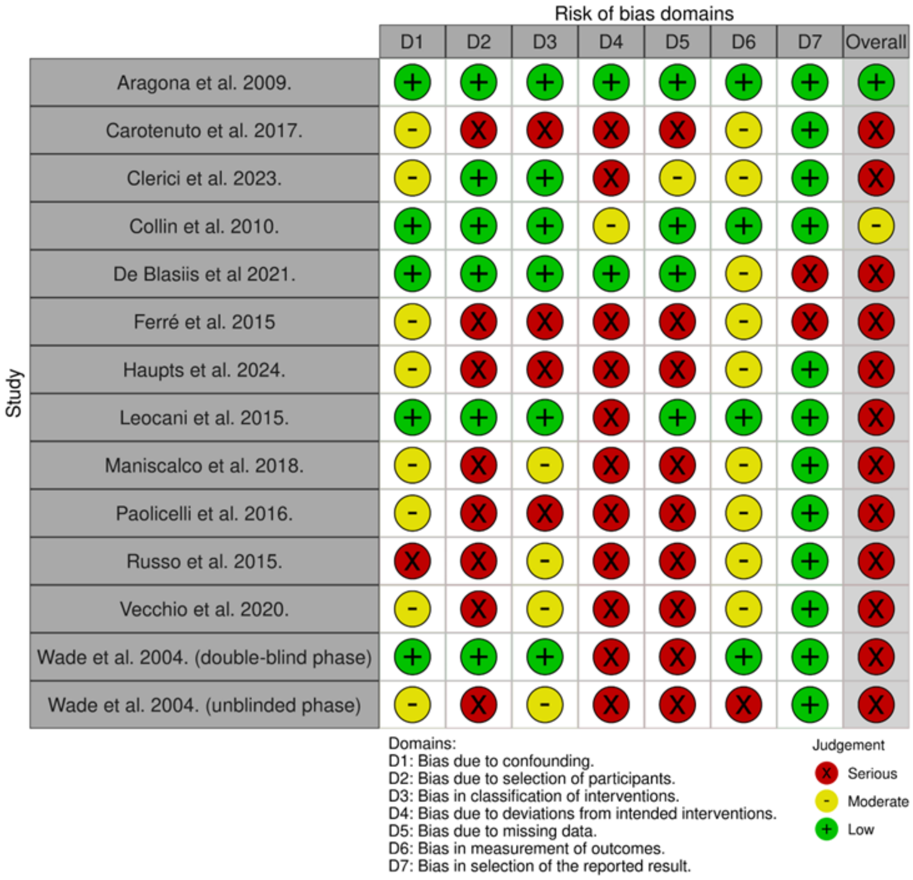

f

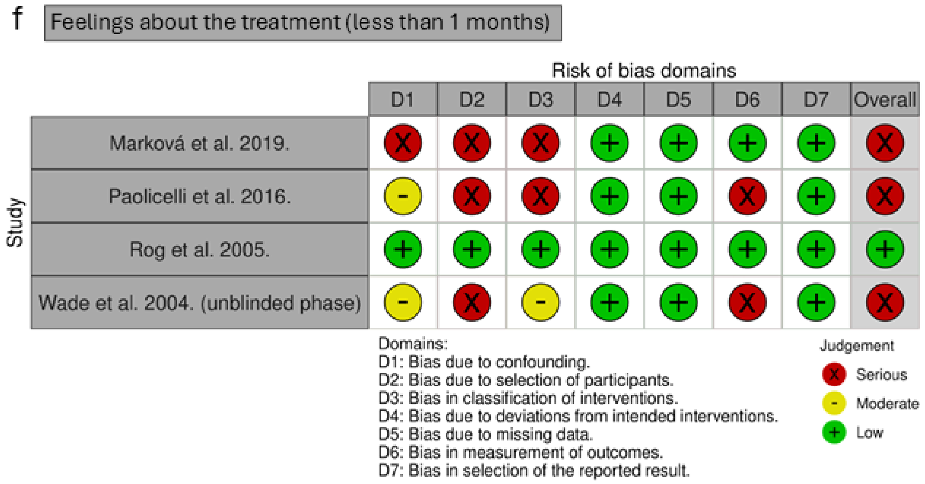
Feelings about the treatment (less than 1 months)
Risk of bias domains
D1 D2 D3 D4 D5 D6 D7 Overall
Study
Marková et al. 2019. X X X + + + + X
Paolicelli et al. 2016. - X X + + X + X
Rog et al. 2005. + + + + + + + +
Wade et al. 2004. (unblinded phase) - X - + + X + X
Domains:
D1: Bias due to confounding.
D2: Bias due to selection of participants.
D3: Bias in classification of interventions.
D4: Bias due to deviations from intended interventions.
D5: Bias due to missing data.
D6: Bias in measurement of outcomes.
D7: Bias in selection of the reported result.
Judgement
Serious
Moderate
Low

g

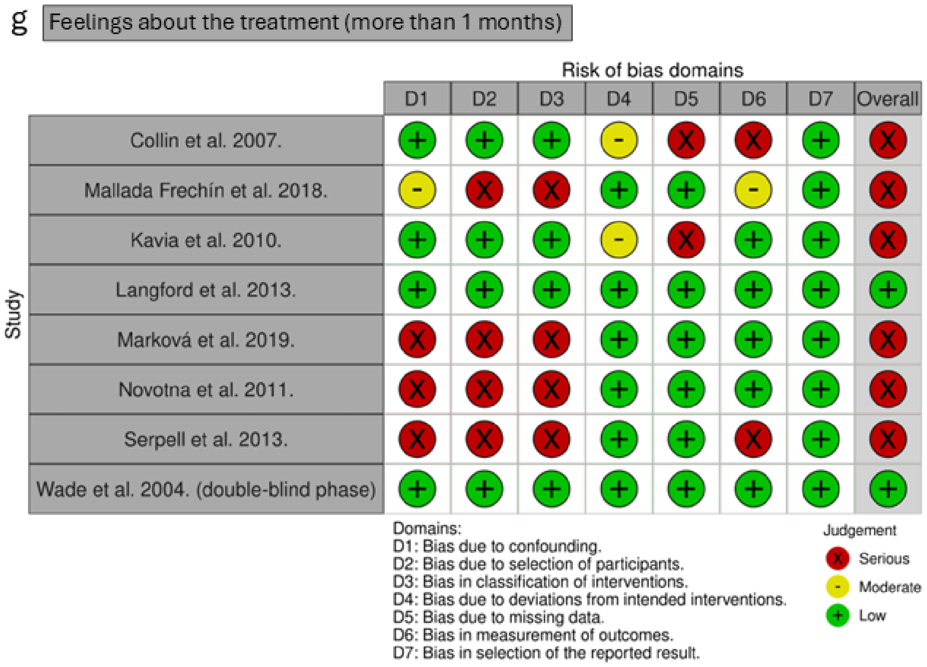
Feelings about the treatment (more than 1 months)
Risk of bias domains
D1 D2 D3 D4 D5 D6 D7 Overall
Study
Collin et al. 2007. + + + - X X + X
Mallada Frechín et al. 2018. - X X + + - + X
Kavia et al. 2010. + + + - X + + X
Langford et al. 2013. + + + + + + + +
Marková et al. 2019. X X X + + + + X
Novotna et al. 2011. X X X + + + + X
Serpell et al. 2013. X X X + + X + X
Wade et al. 2004. (double-blind phase) + + + + + + + +
Domains:
D1: Bias due to confounding.
D2: Bias due to selection of participants.
D3: Bias in classification of interventions.
D4: Bias due to deviations from intended interventions.
D5: Bias due to missing data.
D6: Bias in measurement of outcomes.
D7: Bias in selection of the reported result.
Judgement
Serious
Moderate
Low

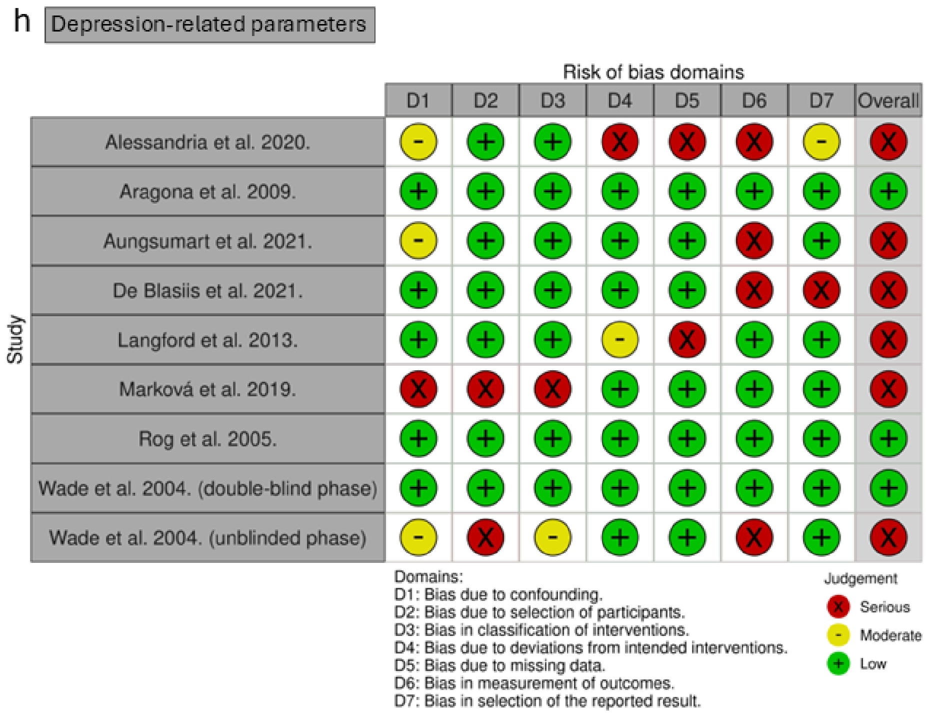

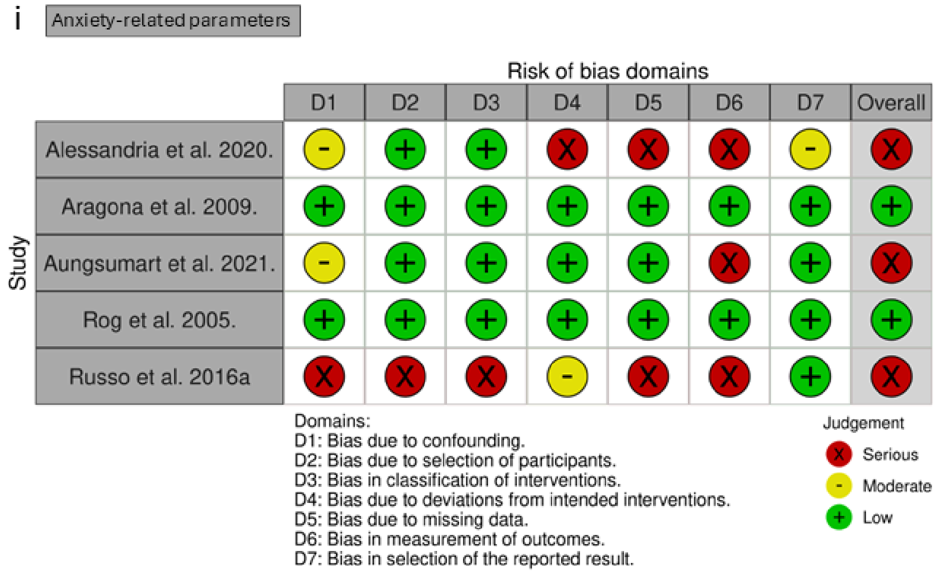

Figure S6

Figure S6. Risk of bias assessment in the collected papers for bladder function (a); sleep disruption (b); spasm quality (c); spasm frequency (d); gait function in timed walk (e); feelings about the treatment (less than a month – f); feelings about the treatment (more than a month – g); depression-related parameters (h) and anxiety-related parameter (i). The ROBINS-I tool was used. Visualization was made with robvis tool.

Supplementary Tables

Table S3

Table S3. Models with lower and higher correlation values for statistical analysis.

Outcome_name	r	k	TE_CI	p_overall	I2	tau2
Bladder function	r0.3	14	-16.15 (-20.73, -11.58)	0,00000377	0,6	41,7291
Bladder function	r0.9	14	-15.83 (-20.34, -11.33)	0,00000396	0,9	55,3109
Sleep disruption	r0.3	17	-19.15 (-24.07, -14.23)	0,000000371	0,8	66,6002
Sleep disruption	r0.9	17	-18.12 (-23.37, -12.87)	0,00000173	1	92,8515
Spasm freqency	r0.3	9	-7.95 (-14.87, -1.03)	0,0294	0,8	65,8
Spasm freqency	r0.9	9	-7.79 (-14.70, -0.88)	0,0316	1	78,2982
Spasm frequency	r0.3	2	-4.65 (-43.63, 34.33)	0,372	0,5	10,5504
Spasm frequency	r0.9	2	-4.26 (-43.64, 35.12)	0,4	0,9	17,9815
Spasm quality	r0.3	9	-21.83 (-30.54, -13.12)	0,000415	0,9	107,2255
Spasm quality	r0.9	9	-20.17 (-29.22, -11.12)	0,000887	1	126,5358
Timed walking (s)	r0.3	22	-6.51 (-11.39, -1.63)	0,0114	1	75,192
Timed walking (s)	r0.9	22	-9.16 (-15.40, -2.92)	0,00604	1	146,611

Table S4

Table S4a. Characteristics of studies included.

Article	Study protocol ID	Study design	Length of study	Number of centres (country)	Qualitatively assessed outcomes	Assessed outcome in meta-analysis
Alessandria et al. 2020	no data	observational	12 months	1 (Italy)	systematic review of anxiety; depressive symptom assessment	
Aragona et al. 2009	no data	randomized, double-blind, placebo-controlled, crossover	3 weeks of treatment + 2 weeks of washout + 3 weeks of treatment + 2 months follow up)	1 (Italy)	systematic review of anxiety; depressive symptom assessment	timed walk
Aungsumart et al. 2021	63029	observational	12 weeks (1 week screening + 4 weeks single blind + max 4 weeks washout + 12 weeks double-blind)	1 (Thailand)	systematic review of anxiety; sleep disruption; spasm quality; depressive symptom assessment;	
Carotenuto et al. 2017	no data	observational	1 year	1 (Italy)		bladder function; sleep disruption; spasm quality; timed walk
Clerici et al. 2023	no data	observational	6 months	not declared (Italy)		bladder function, spasm quality, gait function (timed walk)
Coghe et al. 2015	no data	observational	1 month	1 (Italy)	systematic analysis of gait function	
Collin et al. 2007	GWMS0106	randomized, double blind, parallel group	6 weeks (2 weeks of baseline)	8 (UK); 4 (Romania)		global impression of change
Collin et al. 2010	GWCL0403	randomized, double blind, parallel group	14 weeks (1 week baseline)	15 (UK); 8 Czech Republic		sleep disruption; timed walk
De Blasiis et al. 2021	no data	randomized, unblinded, parallel group	4 weeks	1 (Italy)	depressive symptom assessment	spasm quality; timed walk
Ferrè et al. 2016	no data	observational	48 weeks	1 (Italy)		timed walk
Flachenecker et al. 2014a	MOVE 2 Study (F-2011-030; A 2011 93)	observational	12 months	25 (Germany)	systematic analysis of sleep disruption	
Flachenecker et al. 2014b	MOVE 2 Study (F-2011-030; A 2011 93)	observational	3 months	42 (Germany)	systematic analysis of bladder function	sleep disruption
Guger et al. 2022	no data	observational	3 months	18 (Austria)		sleep disruption, spasm frequency

Table S4b Characteristics of studies included.

Article	Study protocol ID	Study design	Length of study	Number of centres (country)	Qualitatively assessed outcomes	Assessed outcome in meta-analysis
Haupts et al. 2016	GWSP0604 (post hoc analysis of Novotna et al. 2011)	enriched design (single blind then double blind), randomized, parallel group	4 weeks of single-blind + 12 weeks of double-blind + 2 weeks of follow up	18 (UK); 11 (Spain); 10 (Poland); 8 (Czech Republic); 5 (Italy)	systematic analysis of sleep disruption, spasm frequency, gait function (timed walk)	
Haupts et al. 2024	no data	observational	12 weeks	17 (Germany)		bladder function, sleep disruption, gait function (timed walk)
Kavia et al. 2010	GWMS0208 (not present in the original set of hits)	randomized, double-blind, crossover	8 weeks treatment	9 (UK); 3 (Belgium); 3 (Romania)		bladder function; global impression of change
Langford et al. 2013	GWMS0501	2 phase: A.) randomized, double-blind, parallel trial / B.) open plan treatment: randomized-withdrawal study (excluded from analysis)	A.) 14 weeks of randomized, controlled, add-on therapy / B.) 2 weeks of retitration + 12 weeks of stable dose + 4 weeks of randomized-withdrawal phase	12 (UK); 7 (Czech Republic); 5 (Canada); 5 (Spain); 4 (France)	depressive symptom assessment	sleep disruption; global impression of change
Leocani et al. 2015.	M/SATIVX/01	randomized, double-blind, crossover	4 weeks of treatment + 2 week of washout + 4 weeks of treatment	1 (Italy)		sleep disruption; spasm frequency; timed walk
Mallada Frechín et al. 2018	no data	observational	31.9 ± 25.8 months	6 (Spain)		bladder function; sleep disruption; spasm frequency; global impression of change
Maniscalco et al. 2018	no data	observational	31 days (min.: 28; max.: 52)	1 (Italy)		bladder function; timed walk
Marková et al. 2019	H15/02 (SAVANT trial)	enriched design (single blind then double blind), randomized, parallel group	4 weeks of single blind + max 4 weeks of washout + 12 weeks of double-blind	14 (Czech Republic); 1 (Austria)	depressive symptom assessment	sleep disruption; spasm quality; spasm frequency; timed walk; global impression of change

Table S4c Characteristics of studies included.

Article	Study protocol ID	Study design	Length of study	Number of centres (country)	Qualitatively assessed outcomes	Assessed outcome in meta-analysis
Novotna et al. 2011.	GWSP0604	enriched design (single blind then double blind), randomized, parallel group	4 weeks of single-blind + 12 weeks of double-blind + 2 weeks of treatment follow up	18 (UK); 11 (Spain); 10 (Poland); 8 (Czech Republic); 5 (Italy)	depressive symptom assessment; systematic analysis of sleep disruption, spasm frequency, gait function (timed walk)	global impression of change
Paolicelli et al. 2016	no data	observational	40 ± 28 weeks	1 (Italy)		bladder function; timed walk; global impression of change
Patti 2016	Agenzia Italiana del Farmaco (AIFA) government web-based registry	observational	from 1 to 6 months	30 (Italy)	systematic analysis of spasms (not specified); bladder function; sleep disruption	
Patti et al. 2020	37/2015/PO: SA.FE dataset	observational	4 weeks	30 (Italy)	systematic analysis of spasms (not specified); bladder function; sleep disruption; gait function (timed walk)	
Patti et al. 2022.	no data	observational	18 months	32 (Italy)	Systematic review of bladder function, sleep disruption, presence of spasms	
Paul and Silván, 2021	no data	observational	3 months	5 (Germany)		spasm frequency
Rog et al. 2005	GWMS0107	randomized, double-blind, parallel-group	4-week treatment	1 (UK)	systematic review of anxiety; depressive symptoms	sleep disruption; global impression of change
Russo et al. 2015	no data	observational	1 month	1 (Italy)		bladder function; timed walk
Russo et al. 2016	no data	observational	6 months	1 (Italy)	systematic analysis of anxiety	
Sacco et al. 2024	no data	observational	12 weeks	Multicentric (Switzerland)		bladder function
Sartori et al. 2021	no data	observational	1 month	1 (Italy)	systematic analysis of spasm quality	
Serpell et al. 2013	GWMS0106	observational	Mean treatment duration: 334 ± 209 days	8 (UK)	systematic analysis of sleep quality; global impression of change	

Table S4d Characteristics of studies included.

Article	Study protocol ID	Study design	Length of study	Number of centres (country)	Qualitatively assessed outcomes	Assessed outcome in meta-analysis
Vecchio et al. 2020	no data	observational	6 weeks (2 weeks titration + 4 weeks of therapy)	not declared (Italy)		timed walk
Vermersch and Trojano 2016	MOVE-2 EU	observational	3 months	34 (Italy), 2 (Norway), 2 (Denmark)		sleep disruption, spasm frequency
Wade et al. 2004	GWMS0001	GWMS0001: randomized, double-blind, parallel group & GWMS0001 Part B: observational	GWMS0001: 6 weeks & GWMS0001 Part B: 4 weeks	3 (UK)	depressive symptom assessment	bladder function; sleep disruption; spasm quality; timed walk; global impression of change
Wade et al. 2006	GWMS0001 extension	randomized, placebo-controlled, parallel group	remaining on treatment: 434 days (range: 21-814); & stopped: 225 (range: 21-801)	3 (UK)	systematic analysis of bladder function; spasm quality	

Table S5

Table S5a. Patient characteristics.

Publication	Treatment	General daily actuation [N; mean ± SD]	Number of patients	Sex (female% of total)	Age [years; mean ± SD]	Duration of MS [years; mean ± SD]	Baseline spasticity NRS [years; mean ± SD]	Disease phenotype (%)
Alessandria et al. 2020	nabiximols	5 (2-9)[a]	20	55.0	50.2 ± 11.4	20.5 ± 7.2	8.0 (7.0–10.0)[b]	20% RRMS; 70% SPMS; 10% PPMS
Aragona et al. 2009	nabiximols	8.2 ± 3.15	17	64.7	49.8 ± 6.6	20.76 ± 8.42	no data	100% SPMS
	placebo	15.16 + 4.51						
Aungsumart et al. 2021	biosimilar of nabiximols	no data	7	57.1	45.1 ± 7.3	9.6 ± 8.8	6.4 ± 2.3	14.3% RRMS; 71.4% SPMS; 14.3% PPMS
Carotenuto et al. 2017	nabiximols	no data	20	50.0	51.10 ± 9.96	9.21 (2–39)[b]	8.20 ± 1.85	40% RMS; 60% PMS
	no nabiximols treatment	not applicable	10	60.0	39 ± 8.01	4.73 (1–24)[b]	no data	70% RMS; 30% PMS
Clerici et al. 2023	nabiximols	7.7±1.8	31	51.6	51.3 ± 7.9	18.1 ± 7.5	7.2 (6.8–7.6)[p]	51.6% RRMS; 29.0% SPMS; 19.4% PPMS
Coghe et al. 2015	nabiximols	5.6 ± 1.8	20	55.0	49.6 ± 9.11	no data	7.1 ± 1.22	no data
Collin et al. 2007	nabiximols	9.4 ± 6.4	120	64.5	49.7 ± 10.2	13.6 ± 8.6	5.49	no data
	placebo	14.7 ± 8.4	64	52.3	47.8 ± 9.5	12.2 ± 7.7	5.39	no data
Collin et al. 2010	nabiximols	8.5 (1-22)[a]	166 (167 randomized)	63.5	48.0 ± 10.06	14.4 ± 8.29	6.77 ± 0.10[d]	no data
	placebo	15.4 (2-23)[a]	169 (170 randomized)	59.4	47.1 ± 9.15	16.0 ± 8.48	6.48 ± 0.10[d]	no data

Abbreviations: MS: multiple sclerosis; PPMS: primary progressive MS; PRMS: progressive relapsing MS; RRMS: relapsing-remitting MS; SPMS: secondary progressive MS; SD: standard deviation.

Comments: a: (range); b: median (range); c: median; d: standard error was given instead of standard deviation; e: last observed dose; f: no dose-escalation; g: per protocol patients; h: double-blind phase was prioritized; i: data for single-blind phase; j: spasticity related symptoms responders; k: spasticity related symptoms non-responders; l: standard error of mean; m: previously placebo within the 4-week extension; n: previously nabiximols treatment within the 4-week extension; o: calculated from visual analog scale; p: 95% confidence interval; q: upper- and lower quartile

Table S5b Patient characteristics.

Publication	Treatment	General daily actuation [N; mean ± SD]	Number of patients	Sex (female% of total)	Age [years; mean mean ± SD]	Duration of MS [years; mean ± SD]	Baseline spasticity NRS [years; mean ± SD]	Disease phenotype (%)
De Blasiis et al. 2021	nabiximols	3[f]	22	77.3	47.7 ± 10.61	no data	6.64 ± 1.56	45.5% RRMS; 55.5% PMS
	placebo	not applicable	10	50.0	51.9 ± 10.07	no data	6.3 ± 2.26	40.0% RRMS; 60.0% PMS
Ferrè et al. 2016	nabiximols	6.2 ± 2.3	144	52.2	49.7 ± 10.3	17.6 ± 8.5	7.5 ± 1.3	15.3% PPMS; 70.1% SPMS; 14.6 RRMS
Flachenecker et al. 2014a	nabiximols	6.2 ± 2.6	132[g]	55.8	49.4 ± 8.6	14.1 ± 8.0	6.2 ± 1.8	15.4% PPMS; 65.4% SPMS; 19.2% RRMS
Flachenecker et al. 2014b	nabiximols	6.7 ± 2.9[e]	276[g]	60.9	50.0 ± 9.4	15.4±9.0	6.1 ± 1.7	12.3% PPMS; 60.9% SPMS; 26.1% RRMS; 0.7% PRMS
Guger et al. 2023	nabiximols	7.2 ± 3.3[e]	55	60	52.5 ± 9.6	15.1 ± 9.1	6.3±1.6	16.4% RRMS; 32.7% PPMS; 50.9% SPMS;
Haupts et al. 2016	nabiximols	inappropriate data	124	58	49.1 ± 9.1	13.3 ± 8.3	6.8 ± 1.2	no data
	placebo		117	62	48.1 ± 9.6	11.8 ± 8.3	7.0 ± 1.3	no data
Haupts et al. 2024	nabiximols	5.9 ± 3.4[e]	51	60.8	51.4 ± 11.1	14.9 ± 9.8	4.5 ± 1.8	no data
Kavia et al. 2010	nabiximols	8.91	67	72.6	48.6 ± 9.3	no data	no data	no data
	placebo	17.05	68	67.6	46.8 ± 11.2	no data	no data	no data

Abbreviations: MS: multiple sclerosis; PPMS: primary progressive MS; PRMS: progressive relapsing MS; RRMS: relapsing-remitting MS; SPMS: secondary progressive MS; SD: standard deviation.

Comments: a: (range); b: median (range); c: median; d: standard error was given instead of standard deviation; e: last observed dose; f: no dose-escalation; g: per protocol patients; h: double-blind phase was prioritized; i: data for single-blind phase; j: spasticity related symptoms responders; k: spasticity related symptoms non-responders; l: standard error of mean; m: previously placebo within the 4-week extension; n: previously nabiximols treatment within the 4-week extension; o: calculated from visual analog scale; p: 95% confidence interval; q: upper- and lower quartile

Table S5c Patient characteristics.

Publication	Treatment	General daily actuation [N; mean ± SD]	Number of patients	Sex (female% of total)	Age [years; mean ± SD]	Duration of MS [years; mean ± SD]	Baseline spasticity NRS [years; mean ± SD]	Disease phenotype (%)
Langford et al. 2013	nabiximols	8.8 ± 3.87	167	68	48.42 ± 10.43	11.42 ± 8.00	no data	11% PPMS; 39% SPMS; 48% RRMS; 2% PRMS
	placebo	11.1 ± 4.6	172	68	49.51 ± 10.50	12.53 ± 8.50	no data	13% PPMS; 41% SPMS; 45% RRMS; 1% PRMS
Leocani et al. 2015	nabiximols	7 ± 3	34	44	48 ± 7	17.3 ± 8.4	7.1 ± 1.4	100% PMS
	placebo	10 ± 3						
Mallada Frechín et al. 2018	nabiximols	5.6 ± 2.5	32	53.1	52.1 ± 8.6	24.6 ± 22.9	7.8 ± 1.4	9.7% PPMS; 54.8% SPMS; 35.5% RRMS
	no nabiximols treatment	not applicable	10	40.0	52.1 ± 10.9	15.6 ± 15.3	no data	20.0% PPMS; 10.0% SPMS; 70.0% RRMS
Maniscalco et al. 2018	nabiximols	3.8 ± 1.02	15[g]	46.7	56.1 ± 8.6	7.58 (0.3-23.0)	8 (4-10)[b]	no data
Marková et al. 2019[h]	nabiximols	7.3 ± 2.7	53	70.2[i]	51.3 ± 10.2[i]	14.2 ± 8.4[i]	6.4 ± 1.2[i]	11.0% PPMS; 48.2% SPMS; 40.8% RRMS[i]
	placebo	8.5 ± 3.0	53					
Novotna et al. 2011[h]	nabiximols	8.3 ± 2.43	124	58.1	49.1 ± 9.09	13.3 ± 8.29	3.87 ± 1.49	no data
	placebo	8.9 ± 2.31	119	62.4	48.1 ± 9.59	11.8 ± 7.38	3.92 ± 1.55	

Abbreviations: MS: multiple sclerosis; PPMS: primary progressive MS; PRMS: progressive relapsing MS; RRMS: relapsing-remitting MS; SPMS: secondary progressive MS; SD: standard deviation.

Comments: a: (range); b: median (range); c: median; d: standard error was given instead of standard deviation; e: last observed dose; f: no dose-escalation; g: per protocol patients; h: double-blind phase was prioritized; i: data for single-blind phase; j: spasticity related symptoms responders; k: spasticity related symptoms non-responders; l: standard error of mean; m: previously placebo within the 4-week extension; n: previously nabiximols treatment within the 4-week extension; o: calculated from visual analog scale; p: 95% confidence interval; q: upper- and lower quartile

Table S5d Patient characteristics.

Publication	Treatment	General daily actuation [N; mean ± SD]	Number of patients	Sex (female% of total)	Age [years; mean ± SD]	Duration of MS [years; mean ± SD]	Baseline spasticity NRS [years; mean ± SD]	Disease phenotype (%)
Paolicelli et al. 2016.	nabiximols	6.5 ± 1.6	102	49	48.8 ± 10.4	19.2 ± 8	8.7 ± 1.3	24.5% RRMS, 57.8% SPMS, 9.8% PPMS, 7.8% PRMS
Patti 2016	nabiximols	6.2 ± 2.8[e]	1534	52.8	51.0 ± 9.6	17.6 ± 8.6	7.6 ± 1.4	63.7% SPMS; 19.9% RRMS; 16.1% PPMS
Patti et al. 2020.	nabiximols	6.2 ± 2.4[j]<br>6.9 ± 2.5[k]	1432	53.0	50.9 ± 9.7[j]<br>50.9 ± 9.5[k]	18.1 ± 8.9[j]<br>16.8 ± 8.5[k]	8.2 ± 1.4[j]<br>7.6 ± 1.3[k]	84.8% PPMS + SPMS; 15.2% RRMS
Patti et al. 2022.	nabiximols	5.9 ± 2.8[e]	1138	54.6	51.5 ± 9.8	19.8 ± 10.5	7.8 ± 1.25	17.0% RRMS; 66.9% SPMS; 16.1% PPMS
Paul and Silván, 2021	nabiximols	5.2 ± 4.0[e]	22	78,8	48.9 ± 8.9	12.8 ± 6.5	4.0[c]	PPMS: 18.2% , SPMS: 4.5%; RRMS: 77.3% ,
Rog et al. 2005	nabiximols	9.6 ± 6.1	34	82.4	50.3 ± 6.7	10.4 ± 7.3	no data	13.6% PPMS; 50.0% SPMS; 34.8% RRMS; 1.5% benign MS
	placebo	19.1 ± 12.9	32	75.0	48.1 ± 9.7	12.8 ± 8.1	no data	
Russo et al. 2015	nabiximols	no data	37	no data	no data	no data	8.3 ± 0.5	no data
Russo et al. 2016	nabiximols	9	61	no data	42 ± 8.9	9	8 (7-10)[a]	68.9% SPMS; 31.1% RRMS
Sacco et al. 2024.	nabiximols	6 (4-8)[c;q]	95	70	53 (25-86)[b]	17 (2-36)[b]	7 (5-8)[c;q]	46% RRMS; 39% SPMS; 15% PPMS

Abbreviations: MS: multiple sclerosis; PPMS: primary progressive MS; PRMS: progressive relapsing MS; RRMS: relapsing-remitting MS; SPMS: secondary progressive MS; SD: standard deviation.

Comments: a: (range); b: median (range); c: median; d: standard error was given instead of standard deviation; e: last observed dose; f: no dose-escalation; g: per protocol patients; h: double-blind phase was prioritized; i: data for single-blind phase; j: spasticity related symptoms responders; k: spasticity related symptoms non-responders; l: standard error of mean; m: previously placebo within the 4-week extension; n: previously nabiximols treatment within the 4-week extension; o: calculated from visual analog scale; p: 95% confidence interval; q: upper- and lower quartile

Table S5e Patient characteristics.

Publication	Treatment	General daily actuation [N; mean ± SD]	Number of patients	Sex (female% of total)	Age [years; mean mean ± SD]	Duration of MS [years; mean mean ± SD]	Baseline spasticity NRS [years; mean mean ± SD]	Disease phenotype (%)
Sartori et al. 2021	nabiximols	6 (3-11)[b]	36 (both: 11)	50.0%	53.9 ± 8.7	14.83 (0.67 - 37.25)[b]	no data	22.2% PPMS; 52.8% SPMS; 25.0% RRMS
	botulinum toxin	not applicable	28 (both: 11)	46.4%	52.8 ± 10.3	15.63 (4.67 - 39.17)b	no data	17.9% PPMS; 53.6% SPMS; 28.6% RRMS
Serpell et al. 2013	nabiximols	10 ± 8.6	146	64.4%	50 ± 9.0	no data	5.64 ± 0.25[l]	no data
Vecchio et al. 2020	nabiximols	5.5 (3-7)[b]	15	73.3%	55.5 ± 5.2	17.4 ± 6.2	7 (2-10)[b]	100% SPMS
	healthy controls	not applicable	14	71.4%	47.4 ± 5.2	not applicable	not applicable	not applicable
Vermersch and Trojano 2016	nabiximols	6.1[e]	433	55.2%	50.4 ± 10.4	13.7 ± 7.9	6.9 ± 1.9	16.6% PPMS, 51.5% SPMS; 31.6% RRMS
Wade et al. 2004	nabiximols	14.6 ± 1.4[d]; 10.90 ± 0.93[d];[m] / 14.19 ± 1.40[d];[n]	80	65.0%	51.0 ± 9.4	no data	no data	no data
	placebo	23.4 ± 1.8	80	58.8%	50.4 ± 9.3	no data	no data	no data
Wade et al. 2006	nabiximols	10.2	137	60.6%	50.5 (27.5 - 73.8)[a]	no data	6.95 ± 1.47[o]	no data

Abbreviations: MS: multiple sclerosis; PPMS: primary progressive MS; PRMS: progressive relapsing MS; RRMS: relapsing-remitting MS; SPMS: secondary progressive MS; SD: standard deviation.

Comments: a: (range); b: median (range); c: median; d: standard error was given instead of standard deviation; e: last observed dose; f: no dose-escalation; g: per protocol patients; h: double-blind phase was prioritized; i: data for single-blind phase; j: spasticity related symptoms responders; k: spasticity related symptoms non-responders; l: standard error of mean; m: previously placebo within the 4-week extension; n: previously nabiximols treatment within the 4-week extension; o: calculated from visual analog scale; p: 95% confidence interval; q: upper- and lower quartile

Table S6

Table S6. Efficacy of nabiximols treatment on depression and general mental health parameters. Efficacy is separated by its length; cut-off point is one month. Data relates to the longest follow-up in all cases of long-term therapy

Author	Measure	Number of patients (N)	Baseline (mean ± SD)	Data after treatment (mean ± SD)	Difference from baseline (mean ± SD)	Comment
Treatment duration: less than a month						
Aragona et al. 2009.	Symptom Checklist-90 (SCL-90): depression	17	12.76 ± 6.59	15.58 ± 8.65		Lower score indicates better state.
De Blasiis et al. 2021.	Multiple Sclerosis Spasticity Scale: mood	22			-3.58 ± 8.56	Lower score indicates better state.
Marková et al. 2019.	Short form-36: Mental health	53			5.23 (2.19; 8.26)[a]	Higher score indicates better state.
Rog et al. 2005.	Hospital Anxiety and Depression scale for Depression	34			-0.1 ± 2.92	Lower score indicates better state.
Wade et al. 2004.	Beck's Depression Inventory (BDI-II) Score	76			-0.8 ± 7.03	Lower score indicates better state.
Treatment length: more than a month						
Alessandria et al. 2020.	13 item Beck's Depression Invetory	20			0.5	Lower score indicates better state.
Aungsumart et al. 2021.	Depression NRS	5	1 (0; 3)[b]	0 (0; 0)[b]		Lower score indicates better state.
Langford et al. 2013.	Short form-36: Mental health	167			3.17	Higher score indicates better state.

Marková et al. 2019.	Short form-36: Mental health	53	5.52 (2.09; 8.95)[a]	Higher score indicates better state.
Wade et al. 2004	Beck's Depression Inventory (BDI-II) Score	78	-2.1 ± 5.59	Lower score indicates better state.

[a]: least squares mean & 95% confidence interval; [b]: median (lower-; upper quartile)

Table S7

Table S7. Efficacy of nabiximols treatment on anxiety-related parameters. The data in the table are from research that provide exact details on parameters associated to anxiety. Efficacy is separated by its length, cut-off point is one month. Data relates to the longest follow-up in all cases of long-term therapy.

Author	measure	Number of patients (N)	Baseline (mean ± SD)	Data after treatment (mean ± SD)	Difference from baseline (mean ± SD)	comment
Treatment length: less than a month						
Aragona et al. 2009	Self-rating Anxiety Scale	17	36.38 ± 6.42	35.70 ± 7.69		Lower score indicates better state.
Rog et al. 2005	The Hospital Anxiety and Depression Scale score for Anxiety	34			-1.0 ± 2.06	Lower score indicates better state.
Russo et al. 2016a	Zung-sas Scale: Anxiety Index	45	32 ± 1	33 ± 1		Lower score indicates better state.
Treatment length: more than a month						
Alessandria et al. 2020	Hamilton Anxiety Rating Scale	20			1.0	Lower score indicates better state.
Aungsumart et al. 2021	Anxiety Numerical Rating Scale	5	2 (0; 3)a	0 (0;0)a		Lower score indicates better state.
Russo et al. 2016a	Zung-sas Scale: Anxiety Index	45<br>after treatment: 40	32 ± 1	35 ± 10.02		Lower score indicates better state.

[a]: median (lower-; upper quartile)

Table S8

Table S8. Risk of bias assessment of subjects' global impression of change in long-term and short-term cases. The revised Cochrane risk-of-bias tool (RoB2) was used. The assessed features: D1: Randomisation process; D2: Deviations from the intended interventions; D3: Missing outcome data; D4: Measurement of the outcome; D5: Selection of the reported result

Article	Experimental	Comparator	D1	D2	D3	D4	D5	Overall
Subjects' Global Impression of Change (more than a month)								
Collin et al. 2007.	nabiximols	placebo	!	!	+	+	+	!
Kavia et al. 2010.	nabiximols	placebo	+	+	+	+	+	+
Langford et al. 2013.	nabiximols	placebo	+	+	+	+	+	+
Markovà et al. 2019.	nabiximols	placebo	!	+		+	!	
Novotna et al. 2011.	nabiximols	placebo	!	+	+	+	+	!
Wade et al. 2004. (double-blind)	nabiximols	placebo	+	!	+	+	!	!
Subjects' Global Impression of Change (less than a month)								
Markovà et al. 2019.	nabiximols	placebo	!	+	+	+	!	!
Rog et al. 2005.	nabiximols	placebo	+	+	+	+	+	+

+	Low risk
!	Some concerns
	High risk

Table S9

Table S9. Evaluation of evidence level of comparative measures by Grading of Recommendations, Assessment, Development and Evaluations (GRADE) framework.

Certainty assessment							№ of patients		Effect		Certainty	Importance
№ of studies	Study design	Risk of bias	Inconsistency	Indirectness	Imprecision	Other considerations	nabiximols	control	Relative (95% CI)	Absolute (95% CI)		
SGIC												
6	randomised trials	serious	serious[a,b]	serious[c]	not serious	none	180/593 (30.4%)	117/537 (21.8%)	OR 1.69 (1.30 to 2.18)	102 more per 1 000 (from 48 more to 160 more)	⊕◯◯◯ Very low	CRITICAL

Explanations of down-grading: a. Low number of retracted studies. b. The study designs are heterogenous. c. The heterogenous study design may distort the way of participants' answers.

Abbreviations: CI: confidence interval; OR: odds ratio; SGIC: subjects' global impression of change

Table S10

Table S10. Evaluation of evidence level of prognostic measures by Grading of Recommendations, Assessment, Development and Evaluations (GRADE) framework.

№ of studies	Certainty assessment						Effect			Certainty	Importance
	Lowest level study design	Risk of bias	Inconsistency	Indirectness	Imprecision	Other considerations	№ of events	№ of individuals	Rate (95% CI)		
Spasm quality (follow-up: 12 weeks)											
5	non-randomised studies	serious	serious[a,b]	serious[c]	not serious	none	-	190	mean -20.00 (-35.33 to -4.67)	⊕◯◯◯ Very low	CRITICAL
Bladder function (follow-up: 12 weeks)											
8	non-randomised studies	serious	serious[b]	serious[c]	not serious	none	-	302	mean -15.09 (-21.36 to -8.83)	⊕◯◯◯ Very low	CRITICAL
Sleep disruption (follow-up: 12 weeks)											
11	non-randomised studies	serious	serious[b]	serious[c]	not serious	none	-	1085	mean -15.07 (-21.32 to -8.82)	⊕◯◯◯ Very low	CRITICAL
Spasm frequency (follow-up: 12 weeks)											
6	non-randomised studies	serious	serious[a,b]	serious[c]	not serious	none	-	315	mean -4.63 (-11.36 to 2.1)	⊕◯◯◯ Very low	IMPORTANT
Gait (timed walk) (follow-up: 12 weeks)											
13	non-randomised studies	serious	serious[b]	serious[c]	not serious	none	-	502	mean -5.70 (-11.76 to 0.36)	⊕◯◯◯ Very low	IMPORTANT
Subjects' global impression of change (follow-up: range 4 weeks to 12 weeks)											
8	non-randomised studies	serious	serious[b]	serious[c]	not serious	none	235	769		⊕◯◯◯ Very low	IMPORTANT
Subjects' global impression of change (follow-up: 4 weeks)											
4	non-randomised studies	serious	serious[a,b]	serious[c]	serious[d]	none	75	265		⊕◯◯◯ Very low	NOT IMPORTANT
Depression related parameters (follow-up: 4 weeks)											
4	non-randomised studies	serious	serious[a,b]	serious[c]	serious[d]	none	Improvement.			⊕◯◯◯ Very low	NOT IMPORTANT
Depression related parameters (follow-up: range 4 weeks to 12 weeks)											
5	non-randomised studies	serious	serious[a,b]	serious[c]	not serious	none	Improvement.			⊕◯◯◯ Very low	NOT IMPORTANT
Anxiety related parameters (follow-up: 4 weeks)											
3	non-randomised studies	serious	serious[a,b]	serious[c]	serious[d]	none	Improvement.			⊕◯◯◯ Very low	NOT IMPORTANT
Anxiety related parameters (follow-up: range 4 weeks to 12 weeks)											
3	non-randomised studies	serious	serious[a,b]	serious[c]	serious[d]	none	No improvement.			⊕◯◯◯ Very low	NOT IMPORTANT

Explanations of down-grading: a. Low number of retracted studies. b. The study designs are heterogenous. c. Differences in outcomes measures. d. Low number of participants.

Abbreviations: CI: confidence interval
